# Novel risk loci in LGI1-antibody encephalitis: genome-wide association study discovery and validation cohorts

**DOI:** 10.1093/brain/awae349

**Published:** 2024-10-26

**Authors:** Sophie N M Binks, Katherine S Elliott, Sergio Muñiz-Castrillo, Edmund Gilbert, Tânia Kawasaki de Araujo, Andrew R Harper, Andrew C Brown, Amanda Y Chong, Gavin Band, Vicente Peris Sempere, Anne-Laurie Pinto, Felicie Costantino, N William Rayner, Alexander J Mentzer, Norman Delanty, Veronique Rogemond, Géraldine Picard, Adam E Handel, Nico Melzer, Maarten J Titulaer, Soon-Tae Lee, Frank Leypoldt, Gregor Kuhlenbaeumer, Jérôme Honnorat, Emmanuel Mignot, Gianpiero L Cavelleri, Julian C Knight, Sarosh R Irani

**Affiliations:** Oxford Autoimmune Neurology Group, Nuffield Department of Clinical Neurosciences, University of Oxford, Oxford OX3 9DU, UK; Department of Neurology, John Radcliffe Hospital, Oxford OX3 9DU, UK; Centre for Human Genetics, Nuffield Department of Medicine, University of Oxford, Oxford OX3 7BN, UK; Centre for Human Genetics, Nuffield Department of Medicine, University of Oxford, Oxford OX3 7BN, UK; Stanford Center for Sleep Sciences and Medicine, Stanford University, Palo Alto, CA 94304, USA; School of Pharmacy and Biomolecular Sciences, Royal College of Surgeons in Ireland, Dublin, Dublin 2, Ireland; FutureNeuro SFI Research Centre, Royal College of Surgeons in Ireland, Dublin, Dublin 2, Ireland; Department of Medical Genetics and Genomic Medicine, School of Medical Sciences, University of Campinas - UNICAMP, Campinas CEP 13083-888, Brazil; Clinical Development, Research and Early Development, Respiratory and Immunology (R&I), BioPharmaceuticals R&D, AstraZeneca, Cambridge CB2 0AA, UK; Centre for Human Genetics, Nuffield Department of Medicine, University of Oxford, Oxford OX3 7BN, UK; Centre for Human Genetics, Nuffield Department of Medicine, University of Oxford, Oxford OX3 7BN, UK; Centre for Human Genetics, Nuffield Department of Medicine, University of Oxford, Oxford OX3 7BN, UK; Stanford Center for Sleep Sciences and Medicine, Stanford University, Palo Alto, CA 94304, USA; French Reference Centre for Paraneoplastic Neurological Syndromes and Autoimmune Encephalitis, Hospices Civils de Lyon, MeLiS—UCBL-CNRS UMR 5284—INSERM U1314, Université Claude Bernard Lyon 1, Lyon 69008, France; Université Paris-Saclay, UVSQ, INSERM UMR1173, Infection et inflammation, Laboratory of Excellence INFLAMEX, 78180 Montigny-le-Bretonneux, France; Rheumatology Department, APHP, Ambroise Paré Hospital, 92100 Boulogne-Billancourt, France; Centre for Human Genetics, Nuffield Department of Medicine, University of Oxford, Oxford OX3 7BN, UK; Oxford Centre for Diabetes, Endocrinology and Metabolism, Radcliffe Department of Medicine, University of Oxford, Oxford OX3 7LE, UK; Department of Human Genetics, Wellcome Sanger Institute, Hinxton, CB10 1SA, UK; Institute of Translational Genomics, Helmholtz Zentrum München, German Research Center for Environmental Health, D-85764 Neuherberg, Germany; Centre for Human Genetics, Nuffield Department of Medicine, University of Oxford, Oxford OX3 7BN, UK; Nuffield Department of Medicine, University of Oxford, Oxford, OX3 7BN, UK; School of Pharmacy and Biomolecular Sciences, Royal College of Surgeons in Ireland, Dublin, Dublin 2, Ireland; FutureNeuro SFI Research Centre, Royal College of Surgeons in Ireland, Dublin, Dublin 2, Ireland; French Reference Centre for Paraneoplastic Neurological Syndromes and Autoimmune Encephalitis, Hospices Civils de Lyon, MeLiS—UCBL-CNRS UMR 5284—INSERM U1314, Université Claude Bernard Lyon 1, Lyon 69008, France; French Reference Centre for Paraneoplastic Neurological Syndromes and Autoimmune Encephalitis, Hospices Civils de Lyon, MeLiS—UCBL-CNRS UMR 5284—INSERM U1314, Université Claude Bernard Lyon 1, Lyon 69008, France; Oxford Autoimmune Neurology Group, Nuffield Department of Clinical Neurosciences, University of Oxford, Oxford OX3 9DU, UK; Department of Neurology, John Radcliffe Hospital, Oxford OX3 9DU, UK; Department of Neurology, Medical Faculty and University Hospital, Heinrich Heine University Düsseldorf, 40225 Düsseldorf, Germany; Department of Neurology, Erasmus Medical Center, Rotterdam 3015 GD, The Netherlands; Department of Neurology, Seoul National University Hospital, Seoul National University College of Medicine, Seoul 03080, South Korea; Department of Neurology, University Hospital Schleswig-Holstein, 24105 Kiel, Germany; Institute of Clinical Chemistry, University Hospital Schleswig-Holstein, 24105 Kiel, Germany; Department of Neurology, University Hospital Schleswig-Holstein, 24105 Kiel, Germany; French Reference Centre for Paraneoplastic Neurological Syndromes and Autoimmune Encephalitis, Hospices Civils de Lyon, MeLiS—UCBL-CNRS UMR 5284—INSERM U1314, Université Claude Bernard Lyon 1, Lyon 69008, France; Stanford Center for Sleep Sciences and Medicine, Stanford University, Palo Alto, CA 94304, USA; School of Pharmacy and Biomolecular Sciences, Royal College of Surgeons in Ireland, Dublin, Dublin 2, Ireland; Centre for Human Genetics, Nuffield Department of Medicine, University of Oxford, Oxford OX3 7BN, UK; Oxford Autoimmune Neurology Group, Nuffield Department of Clinical Neurosciences, University of Oxford, Oxford OX3 9DU, UK; Department of Neurosciences, Mayo Clinic, Jacksonville, FL 32224, USA; Department of Neurology, Mayo Clinic, Jacksonville, FL 32224, USA

**Keywords:** encephalitis, genetics, GWAS, HLA, LGI1, PTPRD

## Abstract

Encephalitis with antibodies to leucine-rich glioma-inactivated 1 (LGI1-Ab-E) is a common form of autoimmune encephalitis, presenting with seizures and neuropsychiatric changes, predominantly in older males. More than 90% of patients carry the human leukocyte antigen (HLA) class II allele, HLA-DRB1*07:01. However, this is also present in 25% of healthy controls. Therefore, we hypothesized the presence of additional genetic predispositions.

In this genome-wide association study and meta-analysis, we studied a discovery cohort of 131 French LGI1-Ab-E and a validation cohort of 126 American, British and Irish LGI1-Ab-E patients, ancestry-matched to 2613 and 2538 European controls, respectively.

Outside the known major HLA signal, we found two single nucleotide polymorphisms at genome-wide significance (*P* < 5 × 10^−8^), implicating *PTPRD*, a protein tyrosine phosphatase, and *LINC00670*, a non-protein coding RNA gene. Meta-analysis defined four additional non-HLA loci, including the protein coding *COBL* gene. Polygenic risk scores with and without HLA variants proposed a contribution of non-HLA loci. *In silico* network analyses suggested *LGI1* and *PTPRD*-mediated interactions via the established receptors of LGI1, ADAM22 and ADAM23.

Our results identify new genetic loci in LGI1-Ab-E. These findings present opportunities for mechanistic studies and offer potential markers of susceptibility, prognostics and therapeutic responses.

## Teaser

LGI1-antibody encephalitis (LGI1-Ab-E) is a common form of late-onset autoimmune encephalitis. In a genome-wide association study involving 257 patients, Binks *et al.* identify novel non-HLA risk loci with *in silico* plausibility, and a significant overall polygenic disease risk, suggesting a substantial genetic basis to LGI1-Ab-E.

## Introduction

Encephalitis with antibodies to leucine-rich glioma-inactivated 1 (LGI1-Ab-E) is a common autoimmune encephalitis form, presenting with seizures, cognitive deficits and behavioural changes, typically affecting middle-aged and older males.^1^ Its clinical presentation with very frequent seizures is mechanistically underpinned by LGI1's role in modulating K_v_1 channels, and LGI1-antibodies augment downstream neuronal excitability through impact on this pathway.^2,3^ LGI1-Ab-E is overall rare, affecting around one per million in Europe.^4^ The major established genetic risk factor is the human leukocyte antigen (HLA) class II allele HLA-DRB1*07:01.^5-7^ A far smaller role is played by HLA-DRB1*04:02, found in ∼2% ancestry-matched controls.^8^ Since HLA-DRB1*07:01 is carried by ∼90% of LGI1-Ab-E but only ∼25% of non-affected European-ancestry controls, and rare familial LGI1-Ab-E cases have been reported,^9^ we hypothesized additional genetic determinants.^7^

To date, as well as an expected strong HLA peak, a European-ancestry genome-wide association study (GWAS) of 54 LGI1-Ab-E patients found two suggestive (*P* < 1 × 10^−5^) non-HLA signals.^10^ These were rs72961463, close to *DCLK2*, linked to seizures and neuronal migration; and rs62110161, in a little-delineated region of zinc finger genes.^10^ However, this study did not present a validation cohort, a recommended GWAS step.^11^

Here, we focused on extra-HLA genetic associations in the largest-to-date LGI1-Ab-E GWAS, totalling 257 patients, studied as independent discovery and validation cohorts. Beyond the HLA, we identified two replicable signals at genome-wide significance and an additional four by meta-analysis. Our most robust signal implicates the *PTPRD* gene, encoding a hippocampally-expressed protein tyrosine phosphatase, which shares *in silico* networks with LGI1. Furthermore, polygenic risk score (PRS) analyses were compatible with a non-HLA contribution to LGI1-Ab-E genetic risk. Our GWAS is the first that stringently identifies non-HLA genes in LGI1-Ab-E. These findings support future larger GWAS in this and related forms of autoimmune encephalitis to inform disease susceptibility, prognostic factors and therapeutic targets.

## Materials and methods

### Cases, controls and ethical permissions

Patients with serum LGI1-autoantibodies were recruited via tertiary autoimmune neurology centres in Lyon (France; starting cohort *n* = 148), Oxford, UK (*n* = 109), Dublin (Ireland; *n* = 2) and Baltimore/Mayo/San Francisco (USA; *n* = 28). This study was ethically approved in France (Hospices Civils de Lyon; GENDARME, NCT05225883), Ireland (Royal College of Surgeons in Ireland Research Ethics Committee, REC1631) and the UK (Yorkshire & The Humber—Leeds East Research Ethics Committee: 16/YH/0013). USA and Irish patients were consented locally and thereafter recruited to Oxford (total starting cohort *n* = 139; hereafter termed ‘UK recruits’). All patients gave written informed consent.

In total, there were 5151 controls from the UK Biobank (UKBB). UKBB has obtained ethics approval from the North West Multi-centre Research Ethics Committee (approval number: 11/NW/0382) and informed consent from all participants.

### Genome-wide association analysis procedures

#### Array and human leukocyte antigen genotyping

DNA was genotyped on the Axiom Precision Medicine Research Array (French cases), Illumina Global Screening Array v1 or v2 (UK recruits) and a custom Affymetrix chip (UKBB controls).^12^ HLA-DRB allele typing or imputation was available for all cases,^7,8^ and two-digit imputation for controls.^12^ The analysis used Genome Reference Consortium Human Build 37 (hg19) coordinates.

#### Bioinformatics

References for bioinformatics tools and methods used to calculate tissue expression from GTEx data are provided in the Supplementary material, ‘Supplementary Methods’ sections 1 and 2.

##### Quality control procedures

Initial quality control for each cohort was performed in PLINK (v1.9 and v2.0), KING (v2.0.9), flashPCA (v2) and PCAmatchR (v0.3.2). Where chromosome 23 data were available, participants with ambiguous or discrepant sex information were removed, as were individuals or markers with missing data >0.05 and monomorphic single nucleotide polymorphisms (SNPs). The Hardy–Weinberg Equilibrium threshold was <0.00001. For the removal of related individuals, identity-by-descent (IBD) in PLINK and/or KING used a cut-off of 0.185 (PLINK, values between a second- and third-degree relative) or relationship degree 2 (KING).

The quality controlled datasets were passed through a pre-imputation pipeline available at: https://www.strand.org.uk/tools/index.html. Selecting overlapping SNPs only, case/control datasets were merged for principal components analysis [PCA, in PLINK or flashPCA and plotted in R (v4.0.3)]. To address population stratification and retain lambda <1.05, ancestry matching was undertaken with PCAmatchR (v0.3.2). First, French cases were matched 1:20 case:controls from a pool of quality controlled UKBB White European controls (Data-Field 22006), excluding International Statistical Classification of Diseases and Related Health Problems (ICD) codes G04 or G05 (encephalitis). Subsequently, UK-recruited cases were ancestry- and sex-matched 1:20 from remaining UKBB samples, with the same protocol described for French cases.

##### Imputation

Case imputation was performed on the Michigan Imputation Server with the following parameters: rsq filter off, Eagle v2.4 (phased output), reference panel haplotype reference consortium (HRC v1.1). For controls, imputed data were extracted from UKBB. Imputed data were merged as follows: all SNPs with R2 or info score >0.9 were identified, and an intersect list of SNPs-in-common was extracted from each base dataset. Quality control was performed in PLINK as previously outlined, with additional steps of removal of triallelic SNPs and SNPs differentially missing between cases and controls at *P* < 0.01.

##### Association analyses

Discovery and replication association analyses were performed using the PLINK (v1.9) allelic model with a final minor allele frequency (MAF) of 0.01. The independence of signals was assessed with GCTA-COJO (genome-wide complex trait analysis-conditional and joint association analysis) (v1.26.0) using summary statistics prepared in SNPTEST (v2.5.4). A meta-analysis was carried out using GWAMA (v2.1). Clinical analyses were performed in R (v4.0.3).

Suggestive significance was set at *P* < 1 × 10^−5^ and genome-wide significance at *P* < 5 × 10^−8^.

#### Visualization, annotation and *in silico* tools

Manhattan plots were created in ggmanh (v1.7.0) with local genetic architecture visualized with LocusZoom.org. Additional online annotation was achieved with Combined Annotation Dependent Depletion (CADD), GWAS Catalog, GTEx Portal, OMIM, STRING (v12.0), Genemania and WebGestalt (2019).

#### Polygenic risk score

The PRS was calculated using PRSice (v2.3.3)^13^ using a binary phenotype and, due to their slightly larger sample size, the 131 French patients as the target cohort (full parameters are provided in the Supplementary material, ‘Supplementary Methods’ section 4). Analyses were done using the complete dataset, excluding the HLA (chr6:25607979–33607978), restricted to only HLA, excluding chromosome 6 and increasing the MAF to 0.05.

#### Sanger sequencing

Eighty-seven samples from within the UK-recruited validation cohort were Sanger sequenced for the lead *PTPRD* SNP using primers in the Supplementary material, ‘Supplementary Methods’ section 3.

## Results

### Discovery and validation cohorts

We started with 148 French (henceforth, ‘discovery cohort’) and 139 UK-recruited cases (henceforth, ‘validation cohort’). After quality control, our final cohorts numbered 131 and 126, respectively. They did not significantly differ by demographics or clinical features (Table 1) and displayed a classical LGI1-Ab-E phenotype: median age 64–66 years, 30%–31% female, and 95% presenting with encephalitis or seizures. In accordance with established findings,^5-7^ there was an elevated and comparable frequency of HLA-DRB1*07:01 in both cohorts (114/131, 87% and 118/126, 94%, respectively).

**Table 1:** Demographic features of discovery and validation cohorts

	Discovery cohort	Validation cohort	*P* adjusted^a^
**Baseline demographics**			
Number of patients	131	126	na
Median onset age (mean, range)	66 (67, 35–86)	64 (64, 39–90)^b^	0.16534
Female (*n*, %)	39 (30%)	39 (31%)	1
Country of origin (*n*, %)			
France	131 (100%)	0	–
Republic of Ireland	0	2(2%)	–
UK	0	102 (81%)	–
USA	0	22 (17%)	–
**Clinical features**			
CNS	126 (96%)	121 (96%)	1
Limbic encephalitis/epilepsy	125 (95%)	120 (95%)	–
Morvan's syndrome	0	1	–
Other (Miller Fisher-like syndrome)	1	0	–
PNS	3 (2%)	5 (4%)	–
Neuromyotonia	2	3	–
Pain	1	2	–
No details	2 (1.5%)	0	–
Peak mRS median (mean, range)	3 (3.2, 1–6)	3 (3.1, 1–5)	1
**Genetic features**			
HLA DRB1*07:01	114 (87%)	118 (94%)	0.68220
Proportion heterozygous	100 (76%)	101 (80%)	0.78250
Proportion homozygous	14 (11%)	17 (14%)	1

HLA = human leukocyte antigen; mRS = modified Rankin scale; na = not applicable; ns = non-significant.

^a^Holm corrected.

^b^Data on 123 patients available.

**Figure 1:**
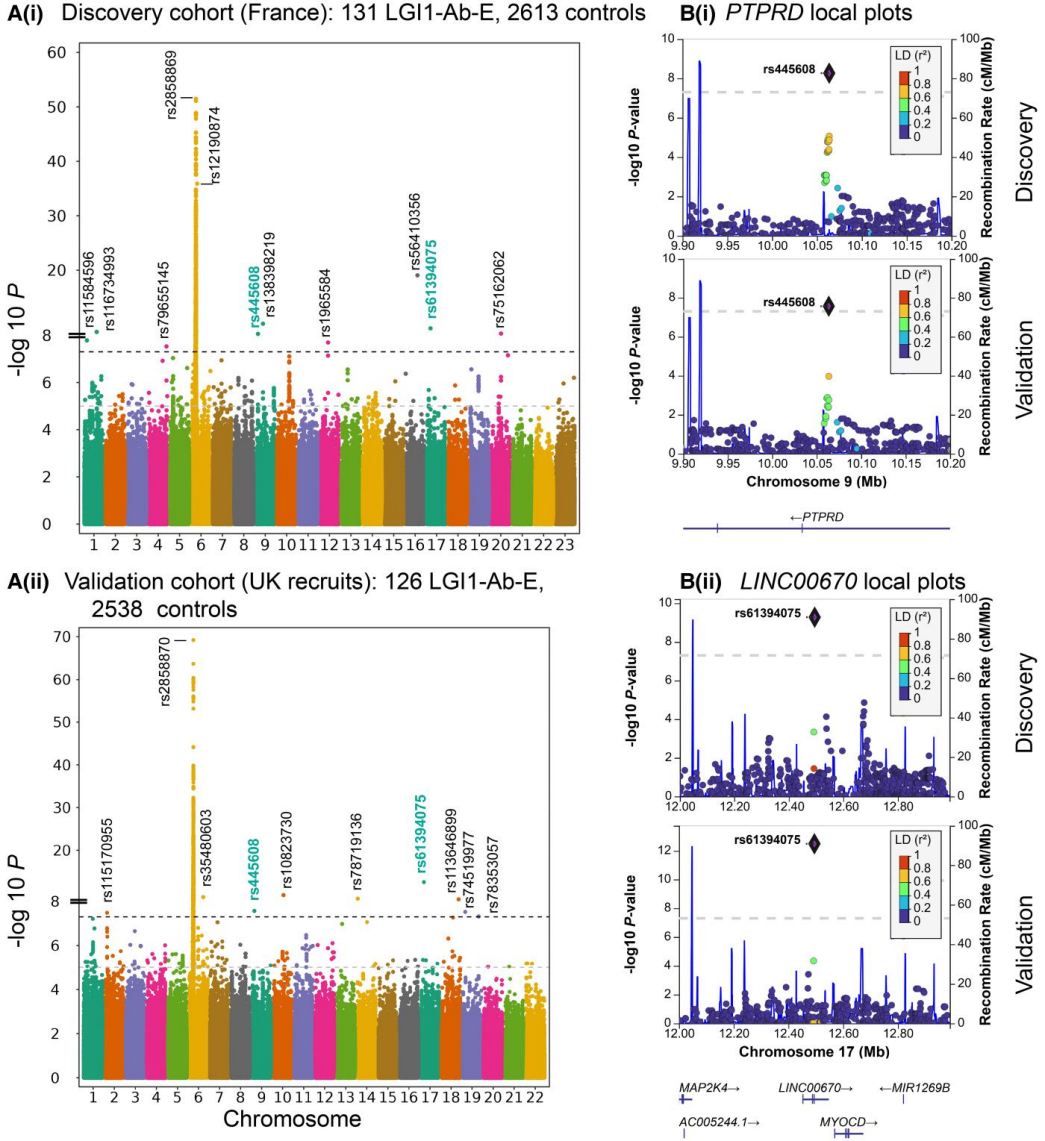
**Manhattan plots and LocusZoom local plots from the main discovery and replication association analyses.** [**A**(**i**)] Manhattan plot of the discovery analysis including 131 French LGI1-Ab-E patients and 2613 matched UK Biobank controls. The analysis included 5 462 363 variants and had a lambda of 1.035. Ten single nucleotide polymorphisms (SNPs) achieving genome-wide significance outside the HLA region and the lead HLA SNP are labelled, with those achieving replication designated in teal labelling. [**A**(**ii**)] Manhattan plot of the validation analysis including 126 White British, Irish and North American patients and 2538 matched UK Biobank controls. The analysis included 5 385 978 variants and had a lambda of 1.015. Nine SNPs achieving genome-wide significance outside the HLA region and the lead HLA SNP are labelled, with those achieving replication designated in teal labelling. Both plots created using ggmanh. The two black bars on the *y*-axes delineate a scale change to accommodate the very low *P*-values associated with the MHC region. The heavy grey dotted line is set at genome-wide significance. [**B**(**i**)] Local LocusZoom plots of genetic architecture of the *PTPRD* hit in the discovery (*top*) and validation (*bottom*) cohorts. [**B**(**ii**)] Local LocusZoom plots of genetic architecture of the *LINC00670* hit in the discovery (*top*) and validation (*bottom*) cohorts. Both plots were created using local linkage disequilibrium data imported from PLINK. The grey dotted line represents genomewide significance. HLA = human leukocyte antigen; *LINC00670* = long intergenic non-protein coding RNA 670; LGI1-Ab-E = leucine-rich glioma-inactivated 1 antibody encephalitis; *PTPRD* = protein tyrosine phosphatase receptor type D.

The final discovery association analysis included 5,462,363 variants across 131 French LGI1-Ab-E (92 male, 39 female), ancestry-matched to 2613 White European controls (957 male, 1656 female; Supplementary Fig. 1). Inflation appeared controlled with a lambda of 1.035 [Fig. 1A(i) and QQ plots in Supplementary Fig. 2]. As expected, there was a strong HLA region signal. The lead HLA SNP, rs2858869, was in tight linkage disequilibrium (LD) (R2 0.997285) with rs28383172, a DRB1*07:01 tag SNP proposed as a genomic marker of HLA-mediated asparaginase hypersensitivity.^14^ Outside the HLA, 10 independent SNPs attained genome-wide significance [Fig. 1A(i), Table 2 and Supplementary Table 1A]. An additional 90 independent non-HLA and 5 HLA SNPs reached at least suggestive significance (Supplementary Table 1A).

**Table 2:** Non-human leukocyte antigen genome-wide significant single nucleotide polymorphisms independent by COJO analysis

SNP^a^	rsID	pval discovery, % effect allele in cases/in controls/OR	pval validation, % effect allele in cases/in controls/OR	pval/direction/OR meta-analysis	Function	CADD^b^ score/annotation	SNP eQTL^c^	Brain specificity/absolute TPM^d^
**Genome-wide significant SNPs from discovery analysis, with validation and meta-analysis results**								
9_10063634_AG Imputed INFO 0.94/Genotyped	rs445608	5.415× 10^−9^**^^ 0.061/0.014 OR: 4.528 (2.60–7.89)	2.73× 10^−8^**^^ 0.052/0.011 OR: 4.876 (2.63–9.04)	2.43× 10^−13^**^/^++^^ OR: 4.68 (3.10–7.07)	*PTPRD* intron variant (between exons 3 and 4)	1.314 Intronic	None	*PTPRD*: 8.02/203.08
17_12495124_AG Imputed INFO 0.94/Imputed INFO 0.96	rs61394075	5.277× 10^−10^**^^ 0.073/0.017 OR: 4.412 (2.65–7.35)	3.192× 10^−13^**^^ 0.091/0.020 OR: 4.898 (3.06–7.85)	3.03× 10^−18^**^/^++^^ OR: 4.67 (3.30–6.60)	*LINC00670* intron variant, *LOC105371540* non-coding transcript variant; upstream of *MYOCD*	1.231 Regulatory/intronic	None	*LINC00670:* 0.0/6.44 *MYOCD:* 0.02/179.39
**Genome-wide significant SNPs in the meta-analysis, attaining at least suggestive and/or nominal significance in discovery and validation cohorts**								
7_51097255_AG Imputed INFO 0.93/Genotyped	rs61739178	0.00136^^^ 0.042/0.016 OR: 2.737 (1.44–5.20)	8.943 × 10^−8^*^^ 0.044/0.009 OR: 5.199 (2.65–10.19)	3.13 × 10^−8^**^/^++^^ OR: 3.71 (2.33–5.91)	COBL (role in skeletal muscle and actin neuron morphogenesis axon/dendrite branching)	16.85 Missense mutation (coding transcripts -p.Ser513Phe) and regulatory feature (non-coding transcript)	None	*COBL*: 0.26/220.62
7_97422926_GT Imputed INFO 0.97/Genotyped	rs1229542	2.354 × 10^−6^*^^ 0.13/0.06 OR: 2.396 (1.65–3.48)	6.571 × 10^−7^*^^ 0.13/0.06 OR: 2.571 (1.75–3.78)	3.36 × 10^−11^**^/^++^^ OR: 2.48 (1.90–3.24)	Intergenic—*TAC1*, *ASNS*	1.108 intergenic	*AC004967.7* (cultured fibroblasts, skeletal muscle, skin)	*TAC1*: 16.10/400.39 *ASNS*: 1.91/779.31
12_130606276_AG Genotyped/Genotyped	rs937529	3.307 × 10^−6^*^^ 0.12/0.05 OR: 2.449 (1.66–3.62)	0.000718^^^ 0.12/0.07 OR: 1.944 (1.31–2.88)	3.06 × 10^−8^**^/^++^^ OR: 2.18 (1.66–2.88)	Intergenic—*TMEM132D* (SCA23, panic disorder), *FZD10*	3.466 intergenic/regulatory	None	*TMEM132D*: 30.97/18.69 *FZD10*: 0.33/42.41
14_21355951_AG Imputed INFO 0.94/Imputed INFO 0.93	rs78719136	0.01668^ 0.038/0.018 OR: 2.207 (1.14–4.29)	2.317 × 10^−9^**^^ 0.056/0.011 OR: 5.161 (2.84–9.39)	2.86 × 10^−8^**^/^++^^ OR: 3.53 (2.26–5.51)	*LOC100507513*, intron variant; in cluster of RNASEs	0.940 intergenic, upstream *RNASE3*	None	*RNASE3*: 0.20/27.72

CADD = Combined Annotation Dependent Depletion; COJO = conditional and joint association analysis; eQTL = expression quantitative trait loci; HLA = human leukocyte antigen; OR = odds ratio; SCA23 = spinocerebellar ataxia 23; SNP = single nucleotide polymorphism; TPM = transcripts per million.

^a^Imputed/genotyped in cases in discovery/replication cohorts. INFO score is given for imputed SNPs, to two decimal places. Scores from Michigan Imputation Server (cases) or UK Biobank imputation scores (controls).

^b^In CADD, a variant in the top 10% of pathogenic variants has a scaled score of 10, in the top 1% has a scaled score of 20.

^c^SNP eQTLs interrogated via GTEx Portal. Accessed 31 December 2023. https://gtexportal.org/home.

^d^Derived from GTEx V6p. For methods see Supplementary material, ‘Supplementary Methods’ section.

^*^Suggestive significance.

^**^Genome-wide significance.

^^^Nominal significance.

^++^Positive direction.

Next, we sought to confirm our findings in a validation cohort of 126 UK-recruited cases (87 male, 39 female), ancestry- and sex-matched to 2538 White European controls (1739 male, 799 female; Supplementary Fig. 3). The validation cohort included 5 385 978 variants with a lambda of 1.015 [Fig. 1A(ii) and QQ plots in Supplementary Fig. 4]. This analysis recapitulated the strong HLA signal in discovery: the lead HLA SNP, rs2858870, was in moderate LD (0.500874) with DRB1*07:01 tag SNP rs2647087^15^ and was the lead HLA signal in the previously published GWAS.^10^ Nine non-HLA SNPs achieved genome-wide significance (Fig. 1B, Table 2 and Supplementary Table 1B). An additional 89 independent non-HLA SNPs attained suggestive significance (Supplementary Table 1B).

Two of the non-HLA SNPs attained genome-wide significance with the same direction in both cohorts (Fig. 1A and B and Table 2). The first, rs445608, is in intron 3 of *PTPRD*, a protein tyrosine phosphatase with dual immune and synaptic actions.^16-18^ The second, rs61394075, lies within intron 3 of the non-protein coding RNA gene *LINC00670*, upstream of the smooth muscle gene *MYOCD*. Local LocusZoom plots using GWAS-specific LD data revealed a supportive pattern for rs445608 [Fig. 1B(i)] but less so for rs61394075 [Fig. 1B(ii)]. In addition, the immune locus, *TRAF3IP2/FYN,* approached replication with different but neighbouring SNPs reaching suggestive significance in each cohort (discovery rs117598088 and validation rs112963264) (Supplementary Table 1 and Supplementary Fig. 5).

### Meta-analysis

Next, we performed a meta-analysis with the software package GWAMA. We only considered variants fulfilling all of: (i) at least nominal significance in one cohort plus suggestive significance in the other; (ii) genome-wide significance in the meta-analysis; (iii) the same directionality of effect in the meta-analysis; and (iv) independent by conditional and joint association analysis program (GCTA-COJO). This stringent analysis identified an additional four hits (Table 2). These included: rs61739178 on chromosome 7, a missense variant in *COBL* with a reported role in neuron morphogenesis and axon/dendrite branching; and rs937529 on chromosome 12, upstream of panic disorder and spinocerebellar ataxia 23 gene *TMEM132D*. Both have potential biological relevance and showed good LD in local plotting profiles (Supplementary Fig. 6A–D). Of all the meta-analysis SNPs, rs61739178 also had the highest deleteriousness rating (CADD score), indicating a top 10% likelihood of pathogenicity (Table 2). The other meta-analysis hits were rs1229542 on chromosome 7 and rs78719136, in a cluster of RNASEs on chromosome 14. Forest plots for all replicated and meta-analysis SNPS are shown in Supplementary Fig. 7A–F.

### Polygenic risk score

**Figure 2:**
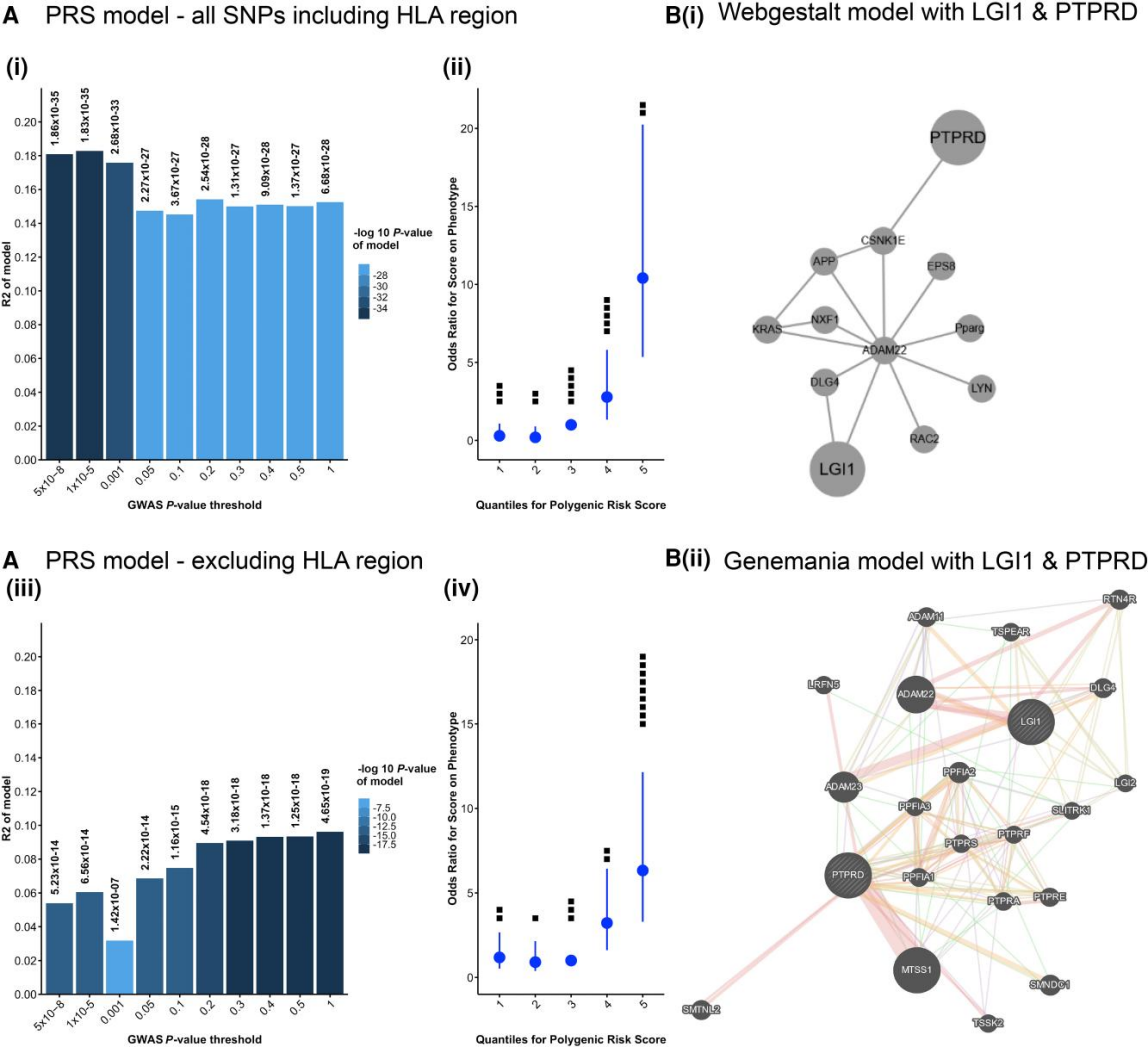
**Polygenic risk scores calculated using PRSice and *in silico* network analyses.** [**A**(**i**–**iv**)] Scores calculated passing in all single nucleotide polymorphisms (SNPs) in chromosomes 1–22. [**A**(**i**)] Polygenic risk scores (PRSs) at different levels of genome-wide association study (GWAS) significance (*x*-axis) and the proportion of the phenotype accounted for by the model (*y*-axis). The significance of each model is shown as the *P*-value at the *top* of each bar. [**A**(**ii**)] Odds ratio of developing the phenotype at each of five quantiles of the PRS. Black dots show numbers of DRB1*07:01-negative patients in each risk quantile. **A**(**iii** and **iv**) show the same plots for the PRS calculated excluding the HLA region from the SNPs passed in excluding the HLA region on chromosome 6 (chr6:25607979–33607978). [**B**(**i**)] *In silico* network analysis created with Webgestalt passing in *LGI1* and *PTPRD* as seed genes. [**B**(**ii**)] *In silico* network analysis created with Genemania passing in *LGI1* and *PTPRD* as seed genes. Pink lines designate physical interactions; lilac, co-expression; light orange, predicted interactions; medium blue, co-localization; green, genetic interactions; and light blue, shared pathways. An intronic variant of *PTPRS* achieved suggestive significance in the discovery cohort. HLA = human leukocyte antigen; LGI1 = leucine-rich glioma-inactivated 1; *PTPRS* = receptor-type tyrosine-protein phosphatase S; *PTPRD* = protein tyrosine phosphatase receptor type D.

Together, these analyses suggested extra-HLA involvement in LGI1-Ab-E. To ask whether combined genetic contributions from all SNPs offer further explanatory power over individually identified SNPs, we employed PRS. In PRS, a genetic risk profile is created using a base GWAS cohort (UK patients), incorporating the effect size of SNPs on a trait in a LD-pruned dataset. The result then bioinformatically predicts the genetic component in a second GWAS (French patients) for the same or a related condition at a user-defined range of *P*-values.^13^ A PRS with all SNPs revealed a significant model at all levels of GWAS significance, with the best-fit model having an R2 (proportion of PRS-assignable phenotypic variance) of 0.18 and a *P*-value of 1.83 × 10^−35^ [Fig. 2A(i)]. In this model, the fifth quantile was significantly enriched for LGI1-Ab-E participants compared to quantiles 1–4 (Supplementary Table 2) and conferred a 10.4 odds ratio of disease for cases versus controls [95% confidence interval (CI) 5.4–20.2; Fig. 2A(ii) and Supplementary Fig. 8]. Reassuringly, with the HLA region (chr6:25607979–33607978) removed, the PRS remained significant at all levels [Fig. 2A(iii)]. The best-fit HLA-depleted model was at a GWAS significance level of 1, with a *P*-value of 4.6 × 10^−19^ and R2 of 0.1, and conferred a LGI1-Ab-E phenotype odds ratio of 6.3 (95% CI 3.3–12.1) [Fig. 2A(iv) and Supplementary Fig. 9]. Moreover, more DRB1*07:01 non-carriers were in the top risk quantile in the HLA-depleted versus the HLA-inclusive model [Fig. 2A(ii and iv)]. These results are compatible with a genetic risk profile with significant HLA and extra-HLA contributions. Further modelling was in alignment with these findings (Supplementary Figs 10–12). Full statistics for all models are in Supplementary Tables 3 and 4.

### *In vitro* and *in silico* confirmation

To confirm our lead signal, rs445608 within *PTPRD* on chromosome 9, found in 13/126 (MAF 0.052) validation patients on the array, we Sanger resequenced 87 individuals with available DNA. This confirmed the effect allele in 8 of 87, conferring a MAF of 0.046 in this sub-group. A further 5/39 carriers lacked DNA for re-sequencing (sub-group MAF, 0.064).

Finally, to create testable molecular hypotheses, we interrogated links between *PTPRD* and *LGI1* using established *in silico* tools: gene set enrichment analysis via WebGestalt and Genemania and protein-protein interactions via STRING. Using *LGI1* and *PTPRD* as seed genes, all methods generated networks linking *PTPRD* and *LGI1* [Fig. 2B(i and ii) and Supplementary Fig. 13]. WebGestalt derived a network including an established key receptor for secreted synaptic LGI1, *ADAM22.* Significant gene ontology pathways by this method focused around glial- and neuro-genesis and synaptic plasticity (Supplementary Table 5), known LGI1 biological functions.^19^ Genemania and STRING showed *PTPRD* at the heart of a network of *PPFIA* genes, a family of LAR protein-tyrosine phosphatase-interacting proteins (liprins), also including another key LGI1-receptor, *ADAM23.*^20^ Other relevant entities depicted included LGI1-interactors *DLG4* and *CASPR2*.

## Discussion

Using a robust approach, with discovery and validation cohorts, we are the first to show replicated extra-HLA hits in LGI1-Ab-E at genome-wide significance. Our most promising hit, rs445608 in *PTPRD*, was supported by favourable LD, is prominent in genetic studies of neurological and neuropsychiatric disease,^18-21^ fear behaviour^22^ and *in silico* analyses delineated close functional links with LGI1. We also showed replication of the imputed SNP rs61394075 in *LINC00670*, a non-coding RNA entity upstream of the smooth muscle gene *MYOCD*. rs61394075 local LD was less supportive; we present it here as this locus was recently implicated in a GWAS of GAD-antibody autoimmunity,^23^ and thus could merit exploration in future cohorts. Our meta-analysis identified four other loci, two with strong biological plausibility. We found no hits involving *LGI1* itself. In addition to individual variants, PRS supports broader non-HLA genetic contributions. Significant models were observed at all levels of GWAS significance, with and without the HLA. The odds ratios are comparable to schizophrenia, well accepted to have a polygenic component,^24^ including in models using the same tool,^24^ and may exceed those for diseases such as coronary artery disease, atrial fibrillation, breast cancer, inflammatory bowel disease, and type 2 diabetes.^25^ Taken together, our findings suggest a complex and substantial genetic architecture in this late-onset illness. Future larger studies should extend these observations, and determine possible initiating roles of environmental factors.

Another subject for further study should be whether *PTPRD*'s role in LGI1-Ab-E predominates through neurological or immune mechanisms. *PTPRD*, like *LGI1*, is a tumour suppressor gene downregulated in glioma^26^ and shapes synapses.^16^ As well as GWAS evidence in restless legs syndrome, epilepsy and schizophrenia,^18,20,21^ it has an immune expression profile, with detection in murine B cell lineages,^16^ and somatic variants as drivers in human marginal zone lymphoma.^17^ Potentially consistent with this dual role, *PTPRD*'s effect in glioma has been linked to interactions with the ‘master transcription factor/cytokine’ STAT3.^26^ While there is no known direct interaction between PTPRD and LGI1, the established role of PTPRD in synapses could reshape these structures,^16^ and either predispose to LGI1 antibody-binding or promote downstream epileptogenesis. Alternatively, indirect interactions via STAT3^26^ and the association of PTPRD with brain volume in neuroinflammation^27^ could suggest an immunomodulatory function.

We also delineated potential links with adaptive immune pathways through locus, but not SNP-specific, replication at *TRAF3IP2/FYN.* This has biological plausibility since *TRAF3IP2* codes for Act1, an adaptor protein with roles in CD40, BAFF and IL-17 signalling, entities with relevance to B cell activation and Th17 pathways, including in our published experimental auto-encoder paradigms.^28,29^

### Limitations

These include the cohort size, nevertheless substantial given LGI1-Ab-E rarity,^4^ population stratification precluding discovery cohort sex-matching and a lack of *in vitro* studies. Despite high PRS odds ratios, the absolute individual risk at the population level, even in the top quintile, would be low. Also, reflecting disease rarity, it is possible our PRS models are over-fitted; further datasets would be required to train the model further. Most variants identified showed low allele frequency in controls (1%–5%), meaning small deviations or imputation inaccuracies could influence results.^30^

## Conclusion

In summary, we have identified novel extra-HLA risk loci and an applicable PRS in LGI1-Ab-E. Our 257 patients were well-phenotyped, and our results suggest our approach could be implemented in other autoimmune encephalitides. The function of disclosed variants should now be investigated *in vitro* and *in vivo*.

## Supplementary Material

awae349_Supplementary_Data

## Data Availability

Qualified investigators with suitable ethics may apply to request the summary statistics via the European Genome-phenome Archive (EGA).
