# Supplementary material for: Novel risk loci in LGI1-antibody encephalitis: genome-wide association study discovery and validation cohorts
Source: Brain. 2024 Oct 26;148(3):737–45. doi: 10.1093/brain/awae349 (PMC11884648; doi:10.1093/brain/awae349)

**Supplementary Table 1: Independent SNPs attaining genomewide or suggestive significance in the discovery or validation cohorts. SNPs in bold were genotyped in cases, those not in bold were imputed In cases, and in loci in red are of potential biological relevance. Additional annotations of interest are given in brackets.**			
**A | Discovery**			
**Lead HLA and independent genomewide SNPs**			
**Chr : pos**	**rsID**	**pval**	**Locus**
1_31333066_AC	rs11584596	1.646e-08	*RP1-65J11.5* non coding transcript variant n.1712C>A
1_176964779_AG	rs116734993	2.384e-09	*ASTN1* intron variant
4_181208791_AT	rs79655145	2.96e-08	Intergenic/cluster of LOC genes
6_32572666_AG	rs2858869	3.371e-52	Lead MHC SNP, upstream of *HLA-DRB1* (not deemed independent by cojo)
6_42454850_AG	rs12190874	1.26e-36	Intergenic
9_36440317_AG	rs138398219	7.514e-11	*RNF38* intron variant
**12_56963259_CT**	rs1965584	2.017e-08	*RBMS2* intron variant
**16_66141709_AC**	rs56410356	8.668e-20	Intergenic, upstream of *CDH5* and *BEAN1*
**20_33276989_GT**	rs75162062	4.816e-09	In cluster of genes; *NCOA6* non coding transcript exon HGVSc n.588C>A and *PIGU* intron HGVSc n.96+9510C>A; SNP eQTLs in *MYH7B*, *EDEM2*, *EIF6* and *PROCR*
**Independent suggestive significance SNPs outside of the HLA region**			
**Chr : pos**	**rsID**	**pval**	**Locus**
1_8458237_CT	rs138941144	5.47e-06	*RERE* intron variant
1_68302223_GT	rs12405521	4.09e-06	*GNG12-AS1* intron variant (*GNG12* regulates *PDL1*)
**1_97789694_AC**	rs59879323	1.03e-06	*DPYD* intron variant
1_114736559_AG	rs12046577	1.60e-06	Intergenic
1_156500096_CT	rs115742679	1.46e-06	*IQGAP3* benign missense variant
1_160106857_AG	rs111331208	3.77e-06	*ATP1A2* intron variant
1_189763978_AC	rs11580958	2.81e-06	*LINC01701* intron variant
1_211826517_AG	rs12724590	6.86e-06	*LINC01693* intron variant
1_227850524_CT	rs139173807	5.48e-07	*ZNF678* intron variant
2_118459069_CG	rs139439100	8.68e-06	Intergenic
2_169387657_CT	rs145607281	3.74e-06	*CERS6* intron variant
2_217418780_AG	rs16856376	3.14e-06	*LOC101928180* intron variant
**3_10551284_CT**	rs12639193	7.10e-06	*ATP2B2* intron variant
3_40283240_CT	rs74967232	1.18e-06	*MYRIP* intron variant
3_52222353_CT	rs73082798	1.25e-06	Intergenic
3_102360572_CT	rs76873706	7.98e-06	*LOC105374016* intron variant
3_150675457_GT	rs60122003	4.94e-06	*CLRN1* intron variant
4_2328398_AG	rs115766033	2.75e-06	*ZFYVE28* intron variant (role in insulin sensitivity)
4_6386881_CT	rs77911941	7.11e-06	*PPP2R2C* intron variant
4_139790911_AT	rs146719745	1.20e-07	*RP11-371F15.3* intron variant
4_188257944_AG	rs72721181	3.81e-06	Intergenic
5_14188667_AG	rs55871001	9.71e-07	*TRIO* intron variant
5_25567343_AT	rs79490934	9.24e-08	Intergenic
5_26012947_AT	rs34318564	8.27e-06	Intergenic
5_26408591_CT	rs7731500	5.36e-07	Intergenic
5_75971257_AG	rs34994307	5.71e-06	*IQGAP2* intron variant
5_81138763_AG	rs56017516	5.32e-06	Intergenic
5_106116159_CT	rs6596705	4.36e-06	Intergenic
5_159218232_AG	rs13173989	8.39e-06	*LINC01847* intron variant
5_162982507_AG	rs139472168	2.44e-07	Intergenic
6_8498718_AG	rs138117954	1.25e-06	Intergenic
6_14138508_AC	rs9396473	6.70e-07	Intergenic, downstream of CD83 dendritic cells
6_16695731_CG	rs150288799	1.74e-07	ATXN1 intron variant
6_88659383_CG	rs16879977	4.03e-06	Intergenic
6_100491783_CT	rs144641472	3.18e-06	MCHR2-AS1 intron variant
6_111915879_AG	rs117598088	2.47e-06	*TRAF3IP2/TRAF31P-AS1* (Th17, BAFF, APRIL pathways)
6_116045903_CG	rs148463024	7.71e-06	Intergenic
6_149210491_CT	rs35738720	2.06e-06	UST intron variant
6_156791461_AG	rs138713767	1.55e-06	Intergenic
6_164527987_AC	rs76882651	5.10e-06	Intergenic
6_170534870_AG	rs67227976	3.26e-07	Intergenic
7_4869782_AT	rs117367818	1.58e-06	*RADIL* intron variant
7_11348766_AG	rs56356722	8.08e-06	*AC004538.3* intron variant; downstream of *THSD7A*
7_33267066_CT	rs111519497	2.05e-06	*BBS9* intron variant
7_45290686_AG	rs143927534	3.90e-06	Intergenic
7_63860350_CT	rs148384528	1.15e-07	Intergenic
7_81409229_AG	rs17429264	6.63e-06	Intergenic, downstream of *CACNA2D1*
7_148950895_CT	rs34185245	1.62e-06	*ZNF212*, missense variant
8_19626760_AC	rs112882827	6.42e-07	Intergenic

8_75671678_CT	rs75717597	6.12e-06	Intergenic
8_75803398_AT	rs72622854	1.58e-06	*RP11-3N13.2* non-coding transcript exon
8_84277394_AG	rs111765531	3.66e-06	Intergenic
**8_102383316_AG**	rs116859215	5.79e-06	Intergenic
8_124805440_CT	rs62519926	6.20e-06	*FAM91A1* intron variant
8_135602421_AC	rs3739426	8.33e-07	*ZFAT* intron variant
9_135376292_CT	rs507144	1.73e-06	*C9orf171* intron variant
10_27331486_CT	rs149035234	8.66e-06	*ANKRD26* intron variant
10_80355051_GT	rs7919676	6.28e-06	LINC00856
10_85773437_GT	rs77967384	1.28e-07	Intergenic
10_115066528_CT	rs3890846	6.15e-06	*LOC105378490* intron variant
**11_36023545_AG**	rs12279993	5.30e-06	*LDLRAD3* intron variant (entry receptor for neuronal damage in Venezuelan Equine Virus)
11_82233433_AG	rs144594481	2.29e-06	*RP11-179A16.1* intron variant
11_100183457_AG	rs150041480	8.28e-06	*CNTN5* intron variant
12_32104564_AG	rs10844065	4.93e-06	Intergenic
13_26081712_AG	rs142377428	9.90e-06	*ATP8A2* (intellectual disability gene) intron variant
13_40339156_AT	rs145532829	8.28e-06	*COG6* intron variant
13_51466164_AG	rs17589481	9.45e-06	*RNASEH2B-AS1* intron variant; *RNASEH2B* implicated in SLE
**13_51845240_AG**	rs75500327	2.80e-07	*FAM124A* intron variant
13_72238922_AT	rs7988451	5.12e-06	*DACH1* intron variant (*DACH1* role in lymphoid development)
13_93009634_AT	rs145736209	6.48e-06	*GPC5* intron variant
**13_101431888_AG**	rs77000308	4.73e-06	*NALCN-AS1* intron variant
14_47562361_CG	rs1041171	4.24e-06	*MDGA2* intron variant
14_67867909_AG	rs111617818	5.71e-06	*GPHN* and *PLEK2* intron variants
14_79006568_CT	rs141662236	2.77e-06	*NRXN3* intron variant
14_85196958_CT	rs151055325	8.51e-06	Intergenic, upstream of *FLRT2*, a netrin receptor interactor
15_46009109_AG	rs117190338	5.04e-06	*LOC105370802* intron variant
**15_69965560_AC**	rs77318289	8.93e-07	*RP11-279F6.3* intron variant
16_305045_AG	rs150541949	4.26e-07	*ITFG3/FAM234A* intron variant
16_48757861_CT	rs62060316	6.02E-06	*LOC105371240* intron variant
17_5762029_CG	rs72479709	2.08e-06	*WSCD1* intron variant
18_31578908_AC	rs146822088	1.34e-06	*NOL4* intron variant
18_47853108_AG	rs149763516	5.32e-06	Intergenic
19_5238512_CG	rs35135733	2.76e-07	*PTPRS* intron variant
19_28966557_CT	rs980016	5.58e-07	*AC005307.3* intron variant
20_21053432_CT	rs6132392	3.95e-06	Intergenic
20_30278704_CT	rs145469972	4.00e-06	*BCL2L1/RP11-243J16.7* intron variant
20_56057703_AG	rs117889718	7.03e-08	Intergenic
X_71404408_CT	rs79635048	1.10e-06	*PIN4* intron variant
**X_149794198_CT**	rs147069190	6.32e-07	*MTM1* intron variant
**Additional HLA SNPs at genomewide significance, deemed independent signals**			
**Chr : pos**	**rsID**	**pval**	**Locus**
**6_31097918_CT**	rs6929464	8.38e-15	*PSORS1C1* intron variant
6_31321753_GT	rs1057387	7.74e-20	*HLA-B* 3' UTR variant
6_32421184_AG	rs28752489	1.56e-35	Intergenic downstream of *HLA-DRA*/upstream *DRB5*
6_32446051_AC	rs17209887	9.17e-32	Intergenic downstream of *HLA-DRA*/upstream *DRB5*
6_33054074_CT	rs9277496	5.98e-06	*HLA-DPB1* intron variant
**B | Validation**			
**Lead HLA and independent genomewide SNPs**			
**Chr : pos**	**rsID**	**pval**	**Locus**
2_24319205_CT	rs115170955	3.302e-08	*FAM228B* non coding transcript variant n.1499C>T
**6_32572251_CT**	rs2858870	6.084e-70	Lead MHC SNP, upstream of HLA-DRB1
6_127737282_CT	rs35480603	1.018e-09	Intergenic - *KIAA0408, SOGA3, C6orf58, AL096711.2*
10_73147307_CT	rs10823730	3.574e-10	Intergenic - *UNC5B, SLC29A3, CDH23*. SNP eQTLs - *SLC29A3* in testis, *CDH23* in tibial nerve
**18_72437428_AG**	rs113646899	3.317e-09	*ZNF407* intronic c.4803-55956A>G
**19_9251304_AG**	rs74519977	3.016e-08	*ZNF317* intronic c.-93+36G>A
**19_51468161_CT**	rs78353057	4.797e-08	*KLK6* intronic c.198-1356G>A
**Independent suggestive significance SNPs outside of the HLA region**			
**Chr : pos**	**rsID**	**pval**	**Locus**
1_20800774_CT	rs12062808	5.49e-06	*LOC124903871* 2KB upstream variant
1_47343053_AG	rs549972541	8.91e-06	*CYP4Z2P* intron variant
1_52646257_AC	rs141588622	5.51e-06	*ZFYVE9* intron variant
1_92164041_GT	rs137985985	2.52e-06	*TGFBR3* intron variant
1_95792935_CT	rs115156977	5.81e-06	Intergenic
1_110508301_CT	rs181520088	6.23e-08	Intergenic
1_112401778_CT	rs67276545	7.13e-07	*KCND3* (potassium channel) intron variant
1_160627411_AG	rs113625742	1.69e-07	*LOC107985220* non coding transcript variant
1_165106504_AG	rs4657401	6.38e-06	*LOC107985452* intron variant
2_29094001_GT	rs191734906	4.01e-07	Intergenic

2_98898006_CT	rs76611993	2.97e-06	*VWA3B* intron variant
**2_195330272_CT**	rs79947094	5.69e-06	*LINC01821*
2_208398117_CG	rs72956023	1.80e-06	*CREB1* intron variant
2_242590099_AG	rs34038381	8.60E-06	*ATG4B* intron variant
3_39500379_AT	rs28379944	9.29e-07	Upstream of *MYRIP*, intergenic, in cluster of genes including *CX3CR1* and *CCR8*
3_66695040_CT	rs115192696	7.61e-06	Intergenic
3_95540757_AG	rs114159235	2.23e-07	Intergenic
3_157840520_CT	rs189355122	1.03e-06	*RSRC1* intron variant
4_2274819_CG	rs148909849	4.70e-06	*ZFYVE28* intron variant (role in insulin sensitivity)
4_8644997_AG	rs4487322	5.89e-06	Intergenic
4_55385931_AG	rs143940877	2.44e-06	Intergenic
4_71960133_CT	rs7690948	3.79e-06	*SLC4A4* intron variant
4_142338503_CT	rs72615906	6.87e-06	Intergenic, upstream of *IL15*
**4_186031823_AG**	rs6552864	9.79e-07	Intergenic
4_187069574_GT	rs142316601	3.76e-06	*FAM149A* intron variant
5_2653777_AG	rs138459225	4.73e-06	Intergenic
5_39392691_AG	rs79744831	2.54e-06	*DAB2* intron variant
5_76253184_CT	rs146015808	2.44e-06	*CRHBP* intron variant
5_110166768_CG	rs146744137	6.48e-06	Intergenic
5_142330890_GT	rs13186446	2.89e-06	*ARHGAP26* intron variant
6_41860313_CT	rs72867173	6.24e-7	*USP49* intron variant
**6_74862615_AC**	rs113429387	9.79e-6	*LOC101928516* : intron variant
6_77980172_CG	rs111372312	1.59e-07	Intergenic
6_83620181_AC	rs80128985	1.54e-06	*UBE3D* (MHC1 protein processing)
6_93532032_CT	rs116685762	9.59e -6	Intergenic
6_111929839_AG	rs112963264	3.56e-07	Intergenic, upstream of *TRAF3IP2*
6_124708100_AG	rs1322586	8.05e-6	*NKAIN2* intron variant
6_161297026_CG	rs77352912	1.50e-06	*RP11-235G24.1/LOC112267969* – intron variant
6_166650171_GT	rs9348099	3.66e-06	*LOC101929297* 2KB upstream variant
7_8987810_GT	rs62447698	6.97e-07	Intergenic, downstream of *NXPH1*
7_11921464_AG	rs76071935	1.85e-06	Intergenic
**7_34484467_CT**	rs76807111	1.09e-06	*NPSR1-AS1* intron variant
**7_50743077_CT**	rs55967814	7.99e-06	*GRB10 intron variant*
**7_97422926_GT**	rs1229542	6.57e-07	Intergenic
7_98146525_AG	rs118105329	1.19e-06	Intergenic upstream of *NPTX2*
7_122766123_AC	rs80094166	6.13e-06	*SLC13A1* intron variant
7_139552588_CG	rs3779134	4.45e-06	*TBXAS1* intron variant
8_66309909_CG	rs75861492	9.22e-07	Intergenic
8_109734599_CT	rs6469199	2.34e-06	*TMEM74* intron variant
**9_138305834_GT**	rs34901388	8.05e-06	Intergenic
10_5873696_AG	rs4749407	8.03e-06	*GDI2*
10_30911733_CT	rs111570772	5.11e-06	*LYZL2* intron variant
10_67690927_CT	rs147834066	2.89e-06	*CTNNA3* intron variant
**10_97168827_CT**	rs11596570	2.21e-06	*SORBS1* intron variant
10_103016127_AC	rs141163442	3.78e-06	*LINC02681* intron variant
**11_15926300_CT**	rs78462694	8.72e-06	Intergenic, downstream of *SOX6*
11_80766301_CT	rs111367719	1.45e-06	Intergenic
11_88012716_AG	rs78924752	4.47e-07	Intergenic
11_91160029_CT	rs16916055	2.39e-06	Intergenic
11_104105973_AG	rs117361561	1.53e-06	Intergenic
11_123767100_CT	rs113666162	1.14e-06	Intergenic
11_133502245_CT	rs143204563	1.04e-06	Intergenic upstream of *OPCML*
**12_17664543_AG**	rs554302918	9.44e-07	*LINC02378/ RP11-871F6.3*
12_92004846_CT	rs111794019	1.31e-06	Intergenic
12_116252880_AG	rs708822	4.07e-06	*LINC02463* intron variant
12_122755192_CT	rs185441718	7.89e-07	Intergenic – cluster of genes
12_130425406_CT	rs112771956	3.47e-06	Intergenic
12_131881442_AG	rs11061502	2.07e-06	Intergenic
13_49457660_AC	rs73184607	1.06e-07	Intergenic
14_64888411_AG	rs145425947	8.76e-08	*MTHFD1* intron variant
14_102004342_CT	rs111264308	6.85e-06	*LOC105370673* intron variant
15_63713500_CG	rs10519209	7.35e-06	*LINC02568* intron variant
15_87199084_AG	rs141263273	4.67e-06	*AGBL1* intron variant
16_52152218_AG	rs118069014	4.95e-06	Intergenic
16_81826366_CT	rs146124551	4.44e-06	*PLCG2* intron variant
16_88951876_CT	rs74739657	8.85e-06	*CBFA2T3* intron variant and *LOC101927793* intron variant
17_8452323_CT	rs7219866	4.47E-06	*MYH10* intron variant
17_21818520_CT	rs138044713	4.65e-06	*LOC105371597* intron variant
17_75068340_AG	rs10221209	6.66e-06	Intergenic
**18_9000042_CT**	rs79541515	5.40e-06	Intergenic

18_32460167_CG	rs62097232	4.87e-07	*DTNA* intron variant
18_49247706_CT	rs150341300	1.83e-06	Intergenic, upstream of *DCC* (Netrin1 receptor)
18_51372285_AG	rs544942247	5.39e-08	Intergenic, downstream of *DCC* (Netrin1 receptor)
18_56649650_CT	rs144617351	2.96e-06	*ZNF532* intron variant
18_74790760_AG	rs60881143	4.80e-06	*MBP intron variant*
20_12998048_AG	rs62201705	9.24e-06	*SPTLC3* intron variant
22_18638657_AG	rs182487538	6.63e-06	*USP18* intron variant (role in type I interferon signalling)
22_20294962_AG	rs113066655	6.48e-06	Intergenic
22_30592915_CT	rs149802821	6.66e-06	*HORMAD2* and *LOC105372988* intron variant
**Additional HLA SNPs at genomewide significance, deemed independent signals**			
**Chr : pos**	**rsID**	**pval**	**Locus**
6_31321753_GT	rs1057387	1.48e-22	*HLA-B* 3' UTR variant
6_32667762_AG	rs2856723	1.60e-19	Intergenic between *HLA-DQA1* and 2
6_32739312_AT	rs6457663	4.65e-30	Intergenic between *HLA-DQB* and *DOB*
6_32976927_AG	rs404557	6.08e-70	*HLA-DOA* intron variant
6_33054074_CT	rs9277496	4.87e-05	*HLA-DPB1* intron variant

**Supplementary Table 2: Fisher/Chisq tests comparing number of LGI1-Ab-E in each quantile, Holm corrected**	
**A | Number of LGI1-Ab-E cases / controls allocated in each Quantile**	
**Q1**	3 / 546
**Q2**	2 / 547
**Q3**	10 / 538
**Q4**	27 / 522
**Q5**	89 / 460
**B | Results of contingency tables**	
**Quantiles compared**	**P-adjusted**
Q1vsQ2	1.0000e+00
Q1vsQ3	1.1164e-01
Q1vsQ4	3.2480e-05**
Q1vsQ5	2.2000e-15**
Q2vsQ3	6.5100e-02
Q2vsQ4	7.3500e-06**
Q2vsQ5	2.2000e-15**
Q3vsQ4	3.0316e-02*
Q3vsQ5	2.2000e-15**
Q4vsQ5	1.4791e-08**
*p<0.05 ** p<0.01. Fisher's exact test used for all tables with a cell with <5 individuals	

**Supplementary Table 3: PRS models at different GWAS significance levels**				
**A | PRS model including the HLA region**				
**Threshold**	**R2**	**P of model**	**Standard Error**	**Number of SNPs in model**
5.00E-08	0.180843	1.86401e-35	0.633223	26
1.00E-05	0.182773	1.83289e-35	1.40803	76
0.001	0.175933	2.68311e-33	155.836	1073
0.05	0.14753	2.27074e-27	12.9613	18346
0.1	0.145212	3.6718e-27	90.3458	31308
0.2	0.154206	2.54085e-28	124.612	52910
0.3	0.149972	1.31347e-27	180.122	71058
0.4	0.151015	9.09347e-28	222.208	86271
0.5	0.150054	1.36628e-27	260.835	98856

1	0.15243	6.67765e-28	291.713	135502
**B | PRS model excluding the HLA region**				
**Threshold**	**R2**	**P of model**	**Standard Error**	**Number of SNPs in model**
5.00E-08	0.0539628	5.23453e-14	0.697821	4
1.00E-05	0.0604717	6.55905e-14	3.39506	37
0.001	0.0318454	1.42255e-07	19.0952	999
0.05	0.0685714	2.22052e-14	92.967	18176
0.1	0.0747662	1.15608e-15	125.833	31088
0.2	0.0895826	4.54126e-18	180.758	52636
0.3	0.0908674	3.18491e-18	223.088	70738
0.4	0.0931821	1.36648e-18	262.34	85909
0.5	0.0934283	1.25039e-18	293.079	98462
1	0.0962455	4.64872e-19	396.853	135021
**C | PRS model including only the HLA region**				
**Threshold**	**R2**	**P of model**	**Standard Error**	**Number of SNPs in model**
5.00E-08	0.172085	5.95882e-34	0.553874	22
1.00E-05	0.159429	7.12438e-32	0.739457	39
0.001	0.162309	2.27686e-32	1.09636	75
0.05	0.151191	2.81951e-30	2.06017	175
0.1	0.15267	1.97269e-30	2.51352	226
0.2	0.152733	2.20031e-30	3.0097	280
0.3	0.148275	1.24132e-29	3.44732	327
0.4	0.149636	8.65941e-30	3.87622	368
0.5	0.148897	9.89765e-30	4.21432	403
1	0.14833	1.21686e-29	5.15492	494
**D | PRS model excluding the whole of chromosome 6**				
**Threshold**	**R2**	**P of model**	**Standard Error**	**Number of SNPs in model**
5.00E-08	0.0488747	2.32628e-13	0.629721	3
1.00E-05	0.0476369	2.24154e-11	3.1887	33
0.001	0.0301051	3.21134e-07	18.6757	930
0.05	0.0635518	1.91917e-13	90.4594	17018
0.1	0.070123	9.28229e-15	123.07	29102
0.2	0.0834939	5.51152e-17	176.40	9378
0.3	0.0852287	3.28018e-17	218.337	66450
0.4	0.0858734	2.65852e-17	256.247	80751
0.5	0.0857949	2.80647e-17	286.77	92565
1	0.0885957	1.01657e-17	387.995	26904
**E | PRS model with MAF of 0.05**				
**Threshold**	**R2**	**P of model**	**Standard Error**	**Number of SNPs in model**
5.00E-08	0.166173	1.27145e-32	0.543191	20
1.00E-05	0.153629	8.36865e-31	0.81421	41
0.001	0.131322	2.18115e-26	8.51964	645
0.05	0.095715	2.58957e-19	72.9537	13258
0.1	0.101995	2.09121e-20	103.258	22283

0.2	0.114965	1.84101e-22	148.03	36245
0.3	0.129548	1.16706e-24	186.674	47727
0.4	0.136258	1.20988e-25	220.372	57305
0.5	0.13938	3.71064e-26	245.034	64735
1	0.143535	7.5003e-27	317.477	84868

**Supplementary Table 4: Risk of phenotype at different quantiles in PRS model**				
**A | PRS model including the HLA region**				
**Quantile**	**OR**	**Upper CI**	**Lower CI**	**Number of individuals**
1	0.296	1.08	0.081	549
2	0.197	0.901	0.043	549
3 (median)	1	1	1	548
4	2.78	5.81	1.33	549
5	10.41	20.25	5.35	549
**B | PRS model excluding the HLA region**				
**Quantile**	**OR**	**Upper CI**	**Lower CI**	**Number of individuals**
1	1.18	2.67	0.526	549
2	0.91	2.15	0.382	549
3 (median)	1	1	1	548
4	3.22	6.43	1.62	549
5	6.33	12.1	3.30	549
**C | PRS model including only the HLA region**				
**Quantile**	**OR**	**Upper CI**	**Lower CI**	**Number of individuals**
1	0.797	2.98	0.213	549
2	0.998	3.47	0.287	549
3 (median)	1	1	1	548
4	7.38	19	2.87	550
5	19.1	47.54	7.68	548
**D | PRS model excluding the whole of chromosome 6**				
**Quantile**	**OR**	**Upper CI**	**Lower CI**	**Number of individuals**
1	0.998	2.17	0.458	549
2	0.841	1.89	0.374	549
3 (median)	1	1	1	548
4	2.38	4.61	1.23	549
5	5.43	9.98	2.95	549
**E | PRS model with MAF of 0.05**				
**Quantile**	**OR**	**Upper CI**	**Lower CI**	**Number of individuals**
1	0.797	2.98	0.212	549
2	0.998	3.47	0.287	549
3 (median)	1	1	1	548
4	5.84	15.2	2.24	549
5	21.0	52.2	8.46	549

Abbreviations: CI, confidence interval; HLA, human leucocyte antigen; OR, odds ratio; PRS, polygenic risk score.

**Supplementary Table 5. Top 10 gene ontology terms in WebGestalt network**	
**Term**	**Adjusted p**
GO:0021782 glial cell development	0.0029
GO:0022008 neurogenesis	0.0029
GO:0048169 regulation of long-term neuronal synaptic plasticity	0.0034
GO:0016601 Rac protein signal transduction	0.0049
GO:0031346 positive regulation of cell projection organization	0.0054
GO:0048468 cell development	0.0084
GO:0048168 regulation of neuronal synaptic plasticity	0.0084
GO:0010001 glial cell differentiation	0.0108
GO:0120036 plasma membrane bounded cell projection organization	0.0109
GO:0007399 nervous system development	0.0109

Supplementary Methods

Supplementary Methods 1: References for bioinformatics tools

Combined Annotation Dependent Depletion (CADD)[1]

flashPCA (v2)[2]

ggmanh (v1.7.0)[3]

GCTA-COJO (v1.26.0)[4]

Genemania[5]

GTEx Portal[6]

GWAMA (v2.1)[7]

GWAS Catalog[8]

KING (v2.0.9)[9]

LocusZoom.org[10]

Michigan Imputation Server[11]

Online Mendelian Inheritance in Man (OMIM)[12]

PLINK (v1.9 and 2.0)[13]*

PCAmatchR (v0.3.2)[14]

qqman(v0.1.9)[15]

SNPTEST (v2.5.4)[16]

STRING (v12.0)[17]

WebGestalt (2019)[18]

References

1. Rentzsch P, Witten D, Cooper GM, Shendure J, Kircher M. CADD: Predicting the deleteriousness of variants throughout the human genome. *Nucleic Acids Res*. 2019;47(D1):D886-D894. doi:10.1093/nar/gky1016

2. Abraham G, Qiu Y, Inouye M. FlashPCA2: principal component analysis of Biobank-scale genotype datasets. Stegle O, ed. *Bioinformatics*. 2017;33(17):2776-2778. doi:10.1093/bioinformatics/btx299

3. Guide to ggmanh package. Accessed December 31, 2023. https://bioconductor.org/packages/devel/bioc/vignettes/ggmanh/inst/doc/ggmanh.html

4. Yang J, Ferreira T, Morris AP, et al. Conditional and joint multiple-SNP analysis of GWAS summary statistics identifies additional variants influencing complex traits. *Nat Genet*. 2012;44(4):369-375. doi:10.1038/ng.2213

5. Mostafavi S, Ray D, Warde-Farley D, Grouios C, Morris Q. GeneMANIA: A real-time multiple association network integration algorithm for predicting gene function. *Genome Biol*. 2008;9(SUPPL. 1):1-15. doi:10.1186/gb-2008-9-s1-s4

6. GTEx Portal. Accessed December 31, 2023. https://gtexportal.org/home

7. Mägi R, Morris AP. GWAMA: software for genome-wide association meta-analysis. *BMC Bioinformatics*. 2010;11(1):288. doi:10.1186/1471-2105-11-288

8. GWAS Catalog. Accessed December 31, 2023. https://www.ebi.ac.uk/gwas/

9. Manichaikul A, Mychaleckyj JC, Rich SS, Daly K, Sale M, Chen W-M. Robust relationship inference in genome-wide association studies. *Bioinformatics*. 2010;26(22):2867-2873. doi:10.1093/bioinformatics/btq559

10. Pruim RJ, Welch RP, Sanna S, et al. LocusZoom: Regional visualization of genome-wide association scan results. *Bioinformatics*. 2011;27(13):2336-2337. doi:10.1093/bioinformatics/btq419

11. Das S, Forer L, Schönherr S, et al. Next-generation genotype imputation service and methods. *Nat Genet*. 2016;48(10):1284-1287. doi:10.1038/ng.3656

12. OMIM. Accessed December 31, 2023. https://www.omim.org/

13. Purcell S, Neale B, Todd-Brown K, et al. PLINK: A Tool Set for Whole-Genome Association and Population-Based Linkage Analyses. *Am J Hum Genet*. 2007;81(3):559-575. doi:10.1086/519795

14. Brown DW, Myers TA, MacHiela MJ. PCAmatchR: A flexible R package for optimal case-control matching using weighted principal components. *Bioinformatics*. 2021;37(8):1178-1181. doi:10.1093/bioinformatics/btaa784

15. D. Turner S. qqman: an R package for visualizing GWAS results using Q-Q and manhattan plots. *J Open Source Softw*. 2018;3(25):731. doi:10.21105/joss.00731

16. Marchini J, Howie B, Myers S, McVean G, Donnelly P. A new multipoint method for genome-wide association studies by imputation of genotypes. *Nat Genet*.

2007;39(7):906-913. doi:10.1038/ng2088

17. STRING. Accessed December 31, 2023. https://string-db.org/

18. Liao Y, Wang J, Jaehnig EJ, Shi Z, Zhang B. WebGestalt 2019: gene set analysis toolkit with revamped UIs and APIs. *Nucleic Acids Res*. 2019;47(W1):W199-W205. doi:10.1093/nar/gkz401

*For additional information on PLINK handling of sex chromosomes see: https://zzz.bwh.harvard.edu/plink/anal.shtml#cc

Supplementary Methods 2: Tissue expression ratios and normalised transcripts per million

The mean expression for each gene across all brain and spinal cord anatomical sites (GTEx V6p, performed using Illumina TruSeq RNA-seq and aligned with TopHat v1.4.1) was calculated. A ratio was obtained by dividing this mean by that obtained for all GTEx tissue sites, including central nervous system sites.

Supplementary Methods 3: Primers

Primers for *PTPRD* SNP 9_10063634_AG (rs445608)

SNP region: 10063951 – 10063424

Forward: AGCTGTGAGGGAAGGTTTTT 20pb, 57.2 ºC, GC:45%

Reverse: AGCAAAAAGAGAAATCTTGGGGT 23pb, 58.8 ºC, GC: 39,1%

Size: 508bp

AGCTGTGAGGGAAGGTTTTTAGTCATAATACTGGCTAATAGTCATGCTCTTTGAACTTTGTAATTTGAGGACTA
TCCACTGGGAGCTAGAGATTCTGAGGTGTTGTTTTTGTTTTTTGTTTTTGTTTTTCCCCAGTACTGCTACCTGCA
ATAGATGTTTCACAGTCCAGTTACCTCTGAACTAGCTGGTATTGCTACATTGCAAGAAATGTGGAGTACTTTTC
ACCTACCCTCATATACCTCAGATTCTATATCTGAATATTATAGGGCTTTCACACCGTAATTTACCAAATTGTGTA
TATTTGTAATCAAGTACATATGACACTAATGTGTTTTGTATTAGAACTGATGATTCTTTTATTACATATGCGGTA
TTCTGCCATAGAGTAGCTATCTGTTCAGTTCAAAGATGTGAAAGCTAAGACTGAAGGAAGTTAAAATATTTCC
AAGGTTTACCAACTAGGTATTTAGATATCTAGATCATAAACCCCAAGATTTCTCTTTTTGCT

Supplementary Methods 4: PRSice parameters

Parameters used for PRSice were as follows:

Rscript PRSice.R --prsice PRSice --a1 A1 --a2 A2 --all-score --bar-levels 5e-08,1e-05,0.001,0.05,0.1,0.2,0.3,0.4,0.5,1 --base validation.assoc --binary-target T --bp BP --chr CHR --extract PRS_snplist.snplist --fastscore --missing set_zero --print-snp --target discovery --thread 11 --quantile 5 --out outfile

Supplementary Results

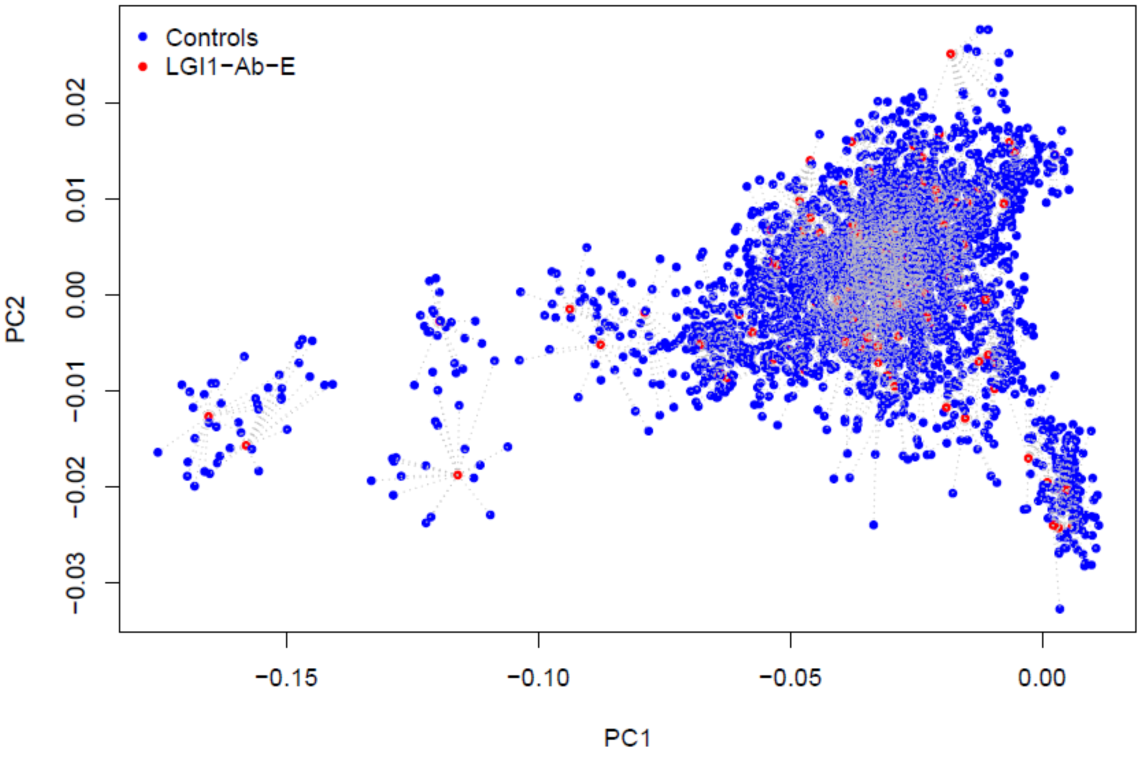

Supplementary Results Figure 1. PCAmatchR plot depicting French LGI1-Ab-E cases (red dots) matched by weighted principal components to 20 each UKBB controls (blue dots). Grey dotted lines connect each case to its allocated controls. **Abbreviations:** LGI1-Ab-E, leucine-rich, glioma-inactivated 1-antibody encephalitis; PC, principal component; UKBB, United Kingdom Biobank.

A

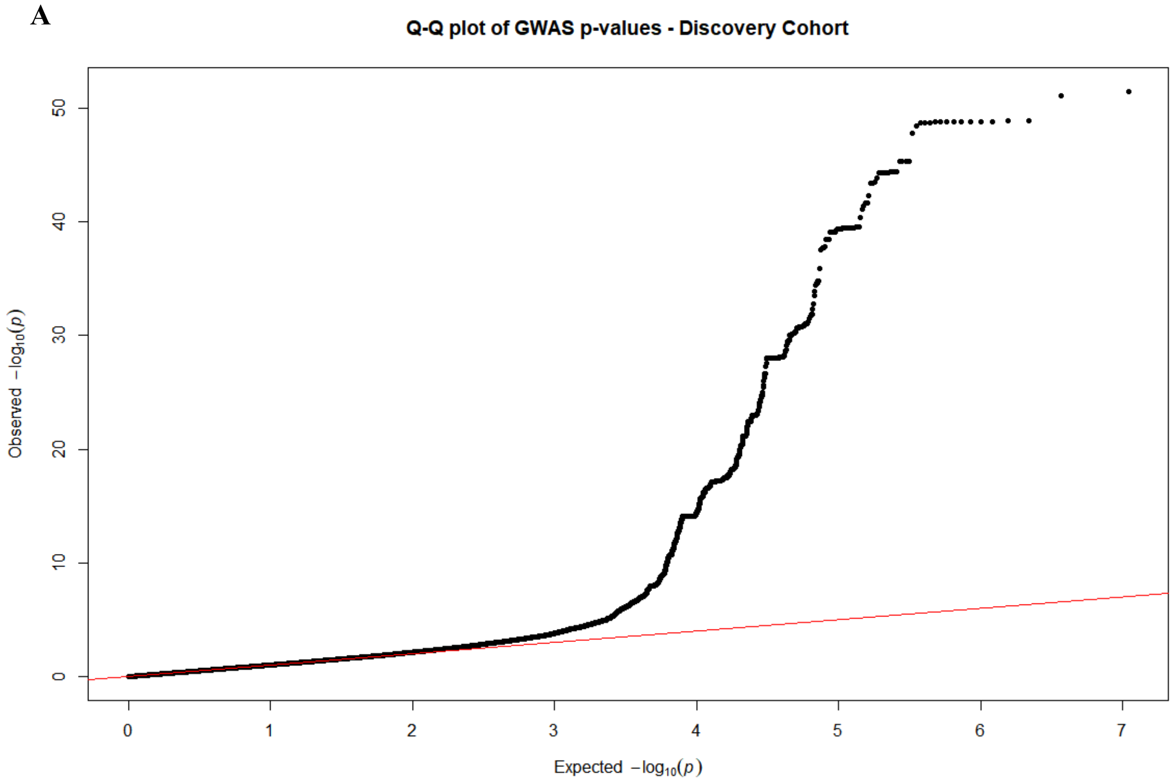

B

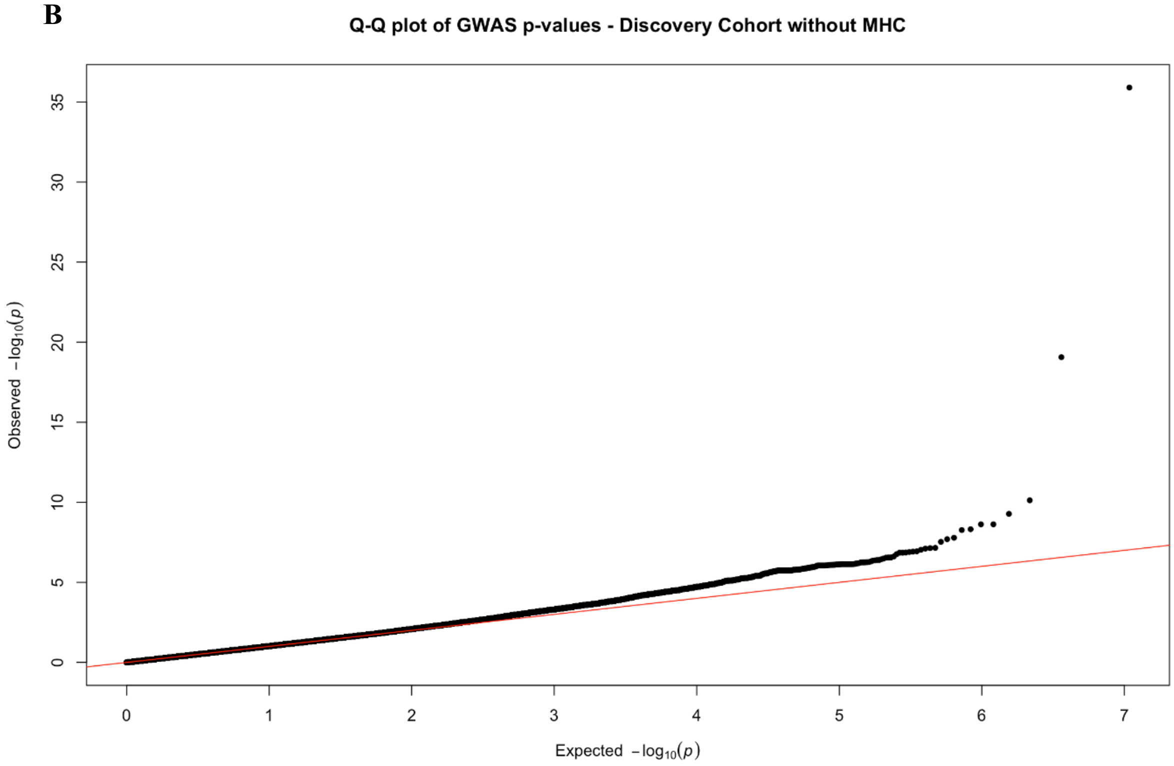

Supplementary Results Figure 2. QQ plots of p-values from the discovery cohort with (A) and without (B) the MHC. Plots generated using qqman (v0.1.9).

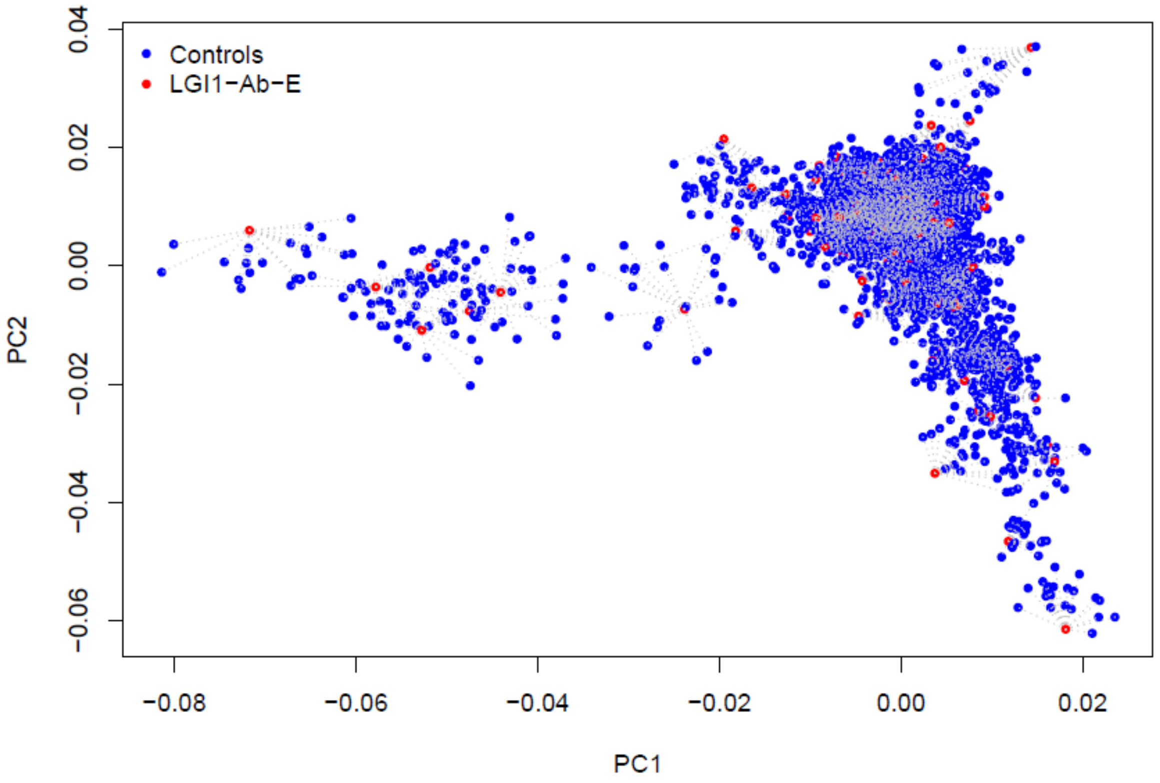

Supplementary Results Figure 3. PCAmatchR plot depicting UK/USA/Irish LGI1-Ab-E cases (red dots) matched by weighted principal components to 20 each UKBB controls (blue dots). Grey dotted lines connect each case to its allocated case. **Abbreviations:** LGI1-Ab-E, leucine-rich, glioma-inactivated 1-antibody encephalitis; PC, principal component; UKBB, United Kingdom Biobank; UK, United Kingdom; US, United States of America

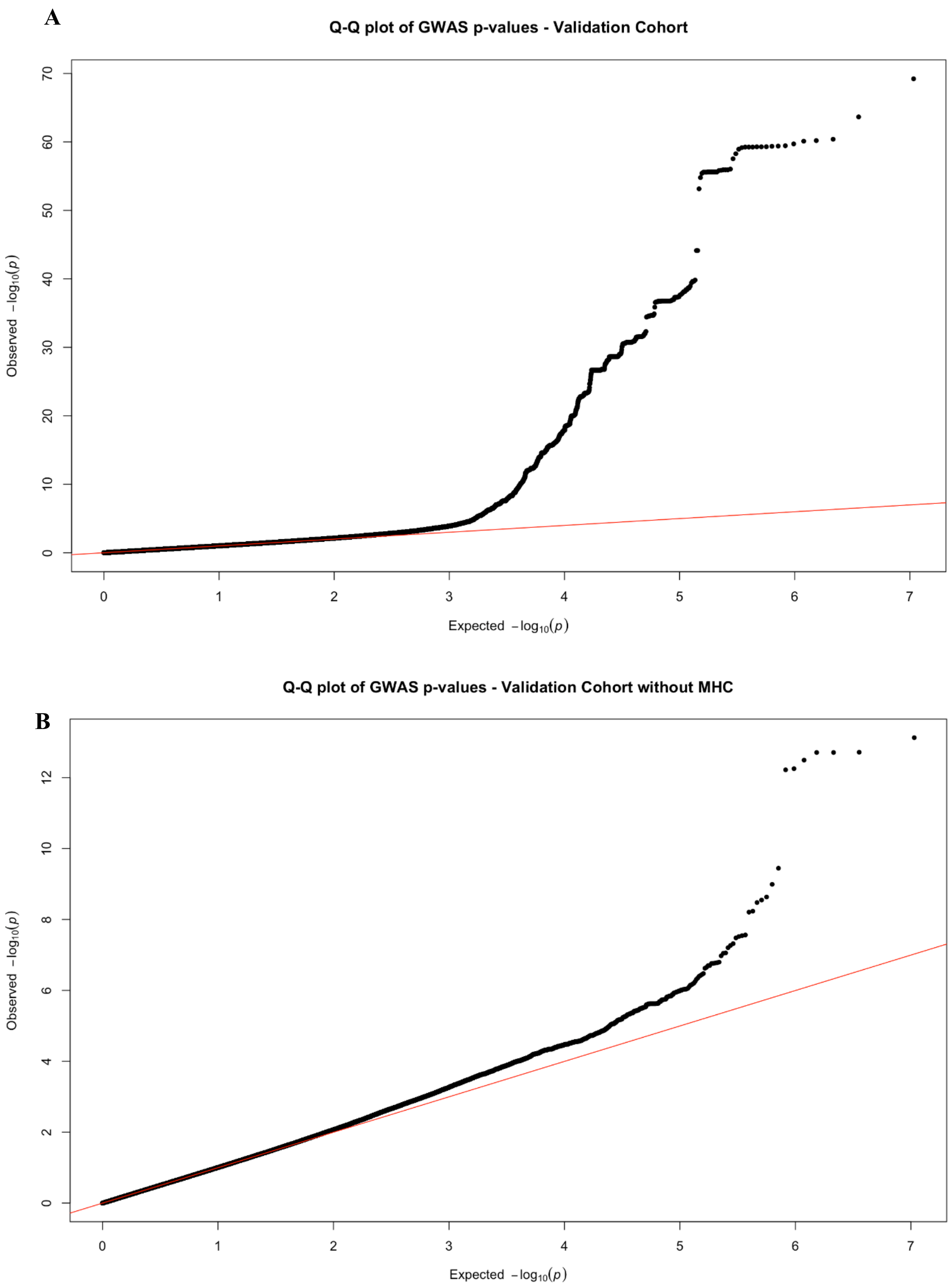

Supplementary Results Figure 4. QQ plots of p-values from the UK recruited cohort with (A) and without (B) the MHC. Plots generated using qqman (v0.1.9).

Supplementary Results Figure 5. LocusZoom local plots for the *TRAF3IP2/FYN* locus. Plots done using LocusZoom European LD.

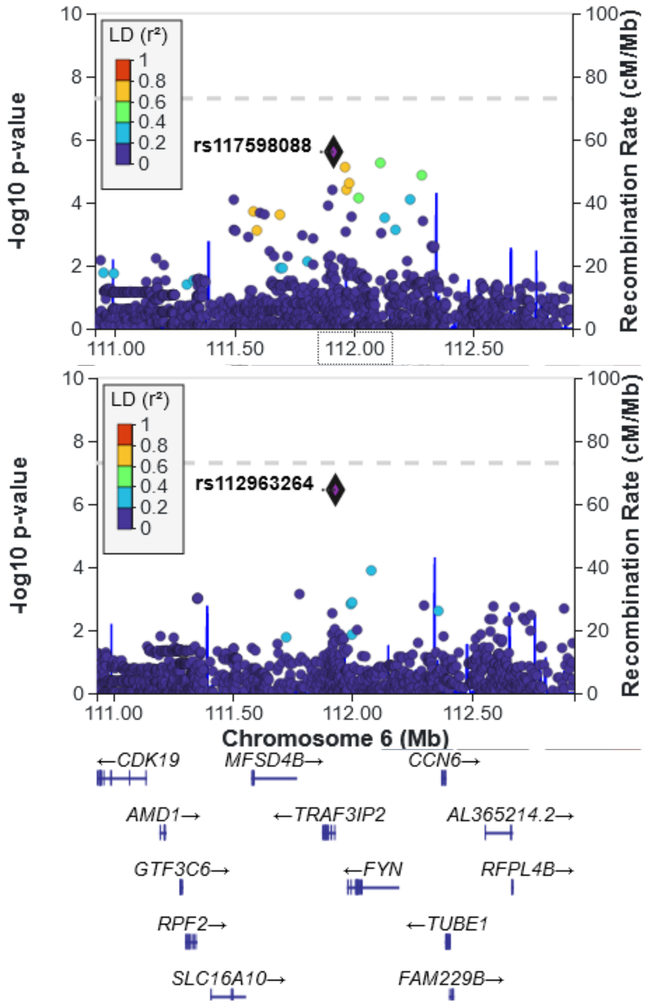

Supplementary Results Figure 6. LocusZoom local plots for the four meta-analysis signals. All plots were done using GWAS-specific LD (linkage disequilibrium) data imported from PLINK.

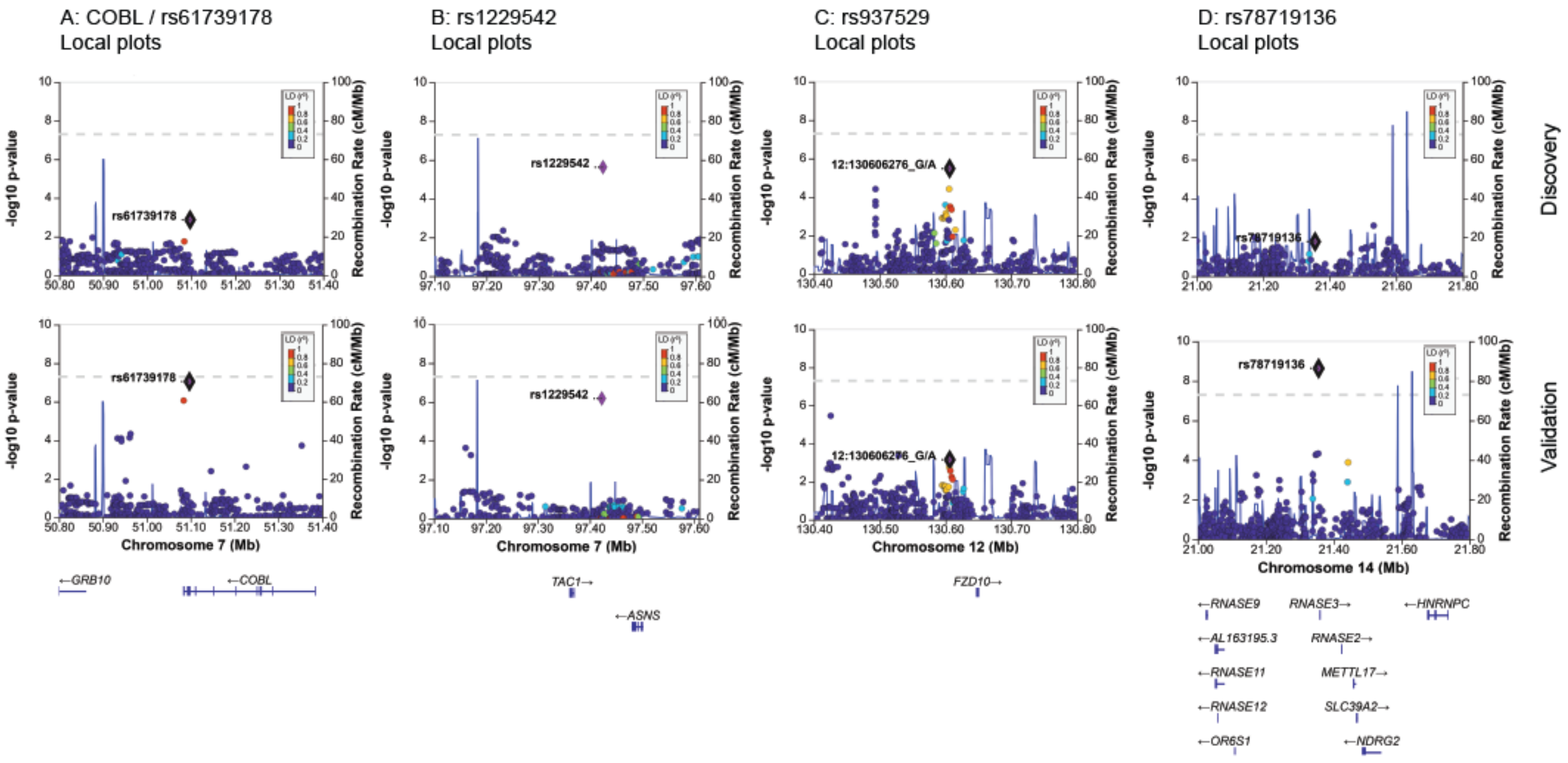

Supplementary Results Figure 7. Forest plots for the two replicated signals (A-B) and four additional meta-analysis signals (C-F), showing odds ratios with 95% lower and upper confidence intervals (CIs): (A) rs445608, *PTPRD* – chromosome 9; (B) rs61394075, *LINC00670* – chromosome 17; (C) rs61739178, *COBL* – chromosome 7; (D) rs1229542 – intergenic, chromosome 7; (E) rs937529, intergenic/*TMEM132D* – chromosome 12; (F) rs78719136, *RNASE3* – chromosome 14.

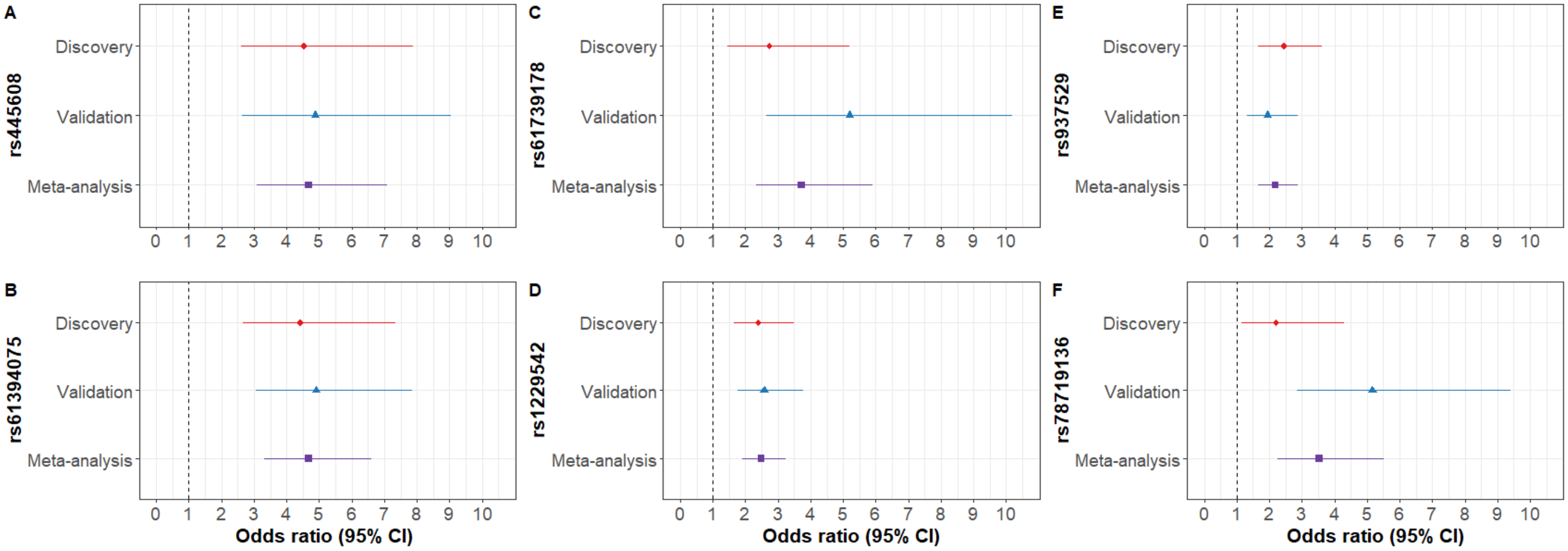

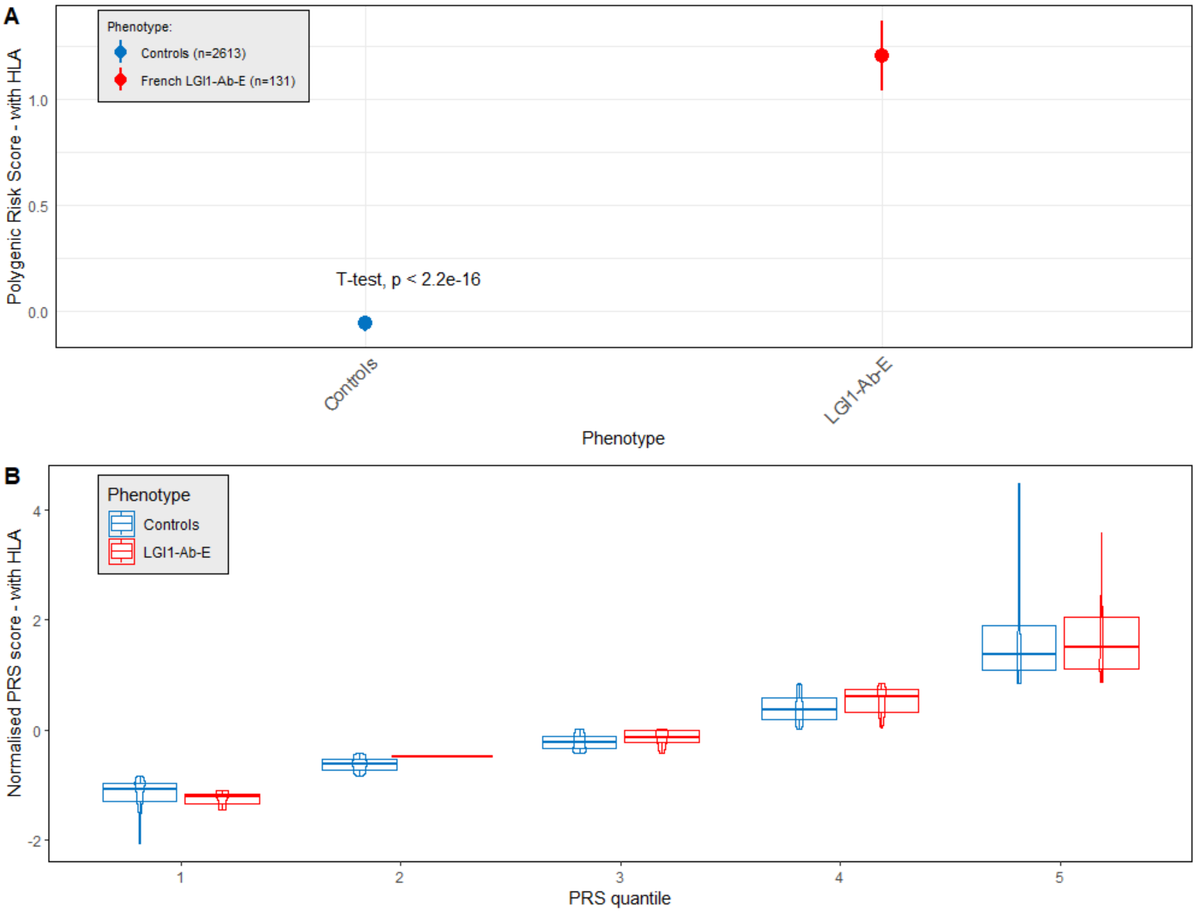

Supplementary Results Figure 8. Polygenic Risk Score, all SNPS including the classical HLA region (additional plots to main Fig. 2A and 2B). Dot plot of mean polygenic risk scores in cases and controls with (A). Scores were normalised to a mean of 0 and SD of 1. (B) Violin plot plotting normalised PRS for controls (blue) and cases (yellow) at each quintile. Horizontal line represents the median value. The number of LGI1-Ab-E in quantile 1-5 was 3 / 2 / 10 / 27 / 89.

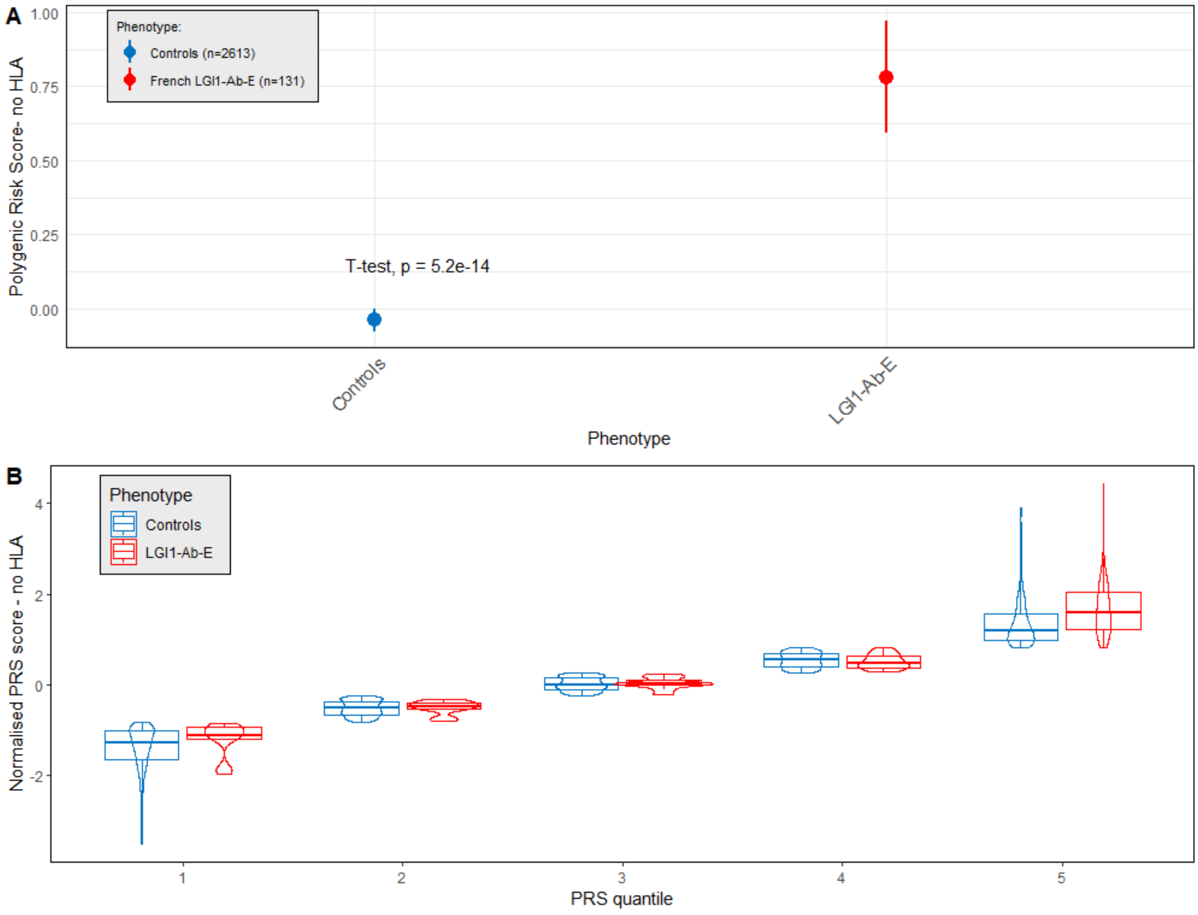

Supplementary Results Figure 9. Polygenic Risk Score excluding the classical HLA region (additional plots to main Fig. 2C and D). Dot plot of mean polygenic risk scores in cases and controls with (A). Scores were normalised to a mean of 0 and SD of 1. (B) Violin plot plotting normalised PRS for controls (blue) and cases (yellow) at each quintile. Horizontal line represents the median value. The number of LGI1-Ab-E in quantile 1-5 was 13 / 10 / 11 / 34 / 63.

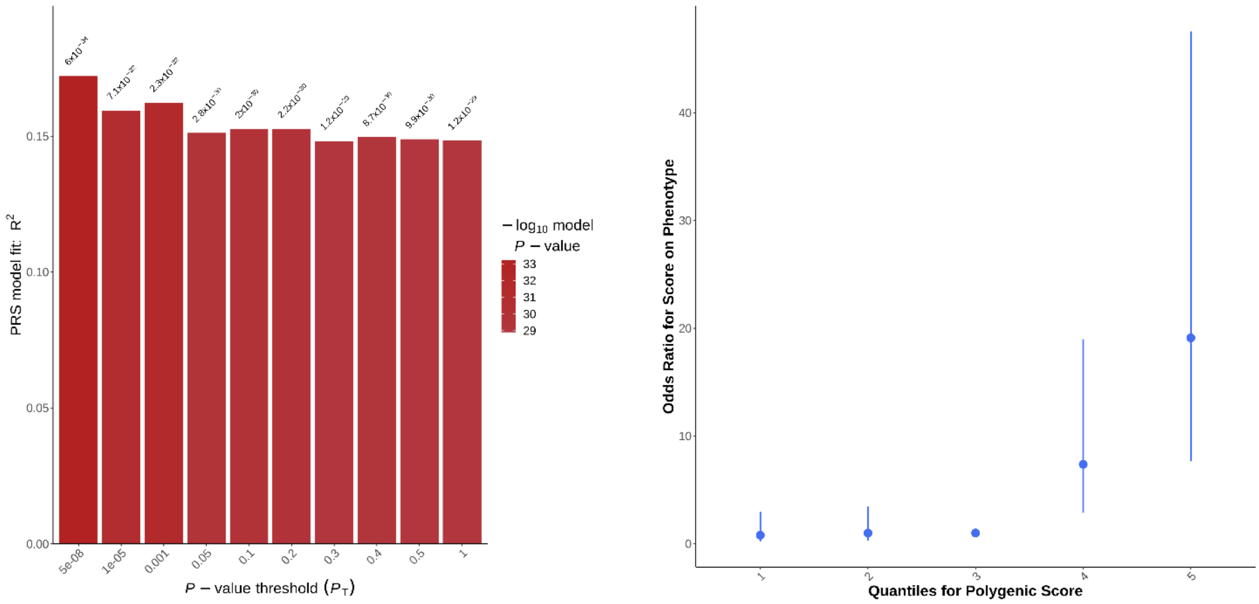

Supplementary Results Figure 10. Polygenic Risk Score Model - only the HLA region. Bar chart x-axis shows the PRS at different levels of GWAS significance; y-axis shows proportion of the phenotype accounted for by the model. The significance of each model is shown on top of each bar. Dot plot shows the odds ratio of developing the phenotype at each quantile.

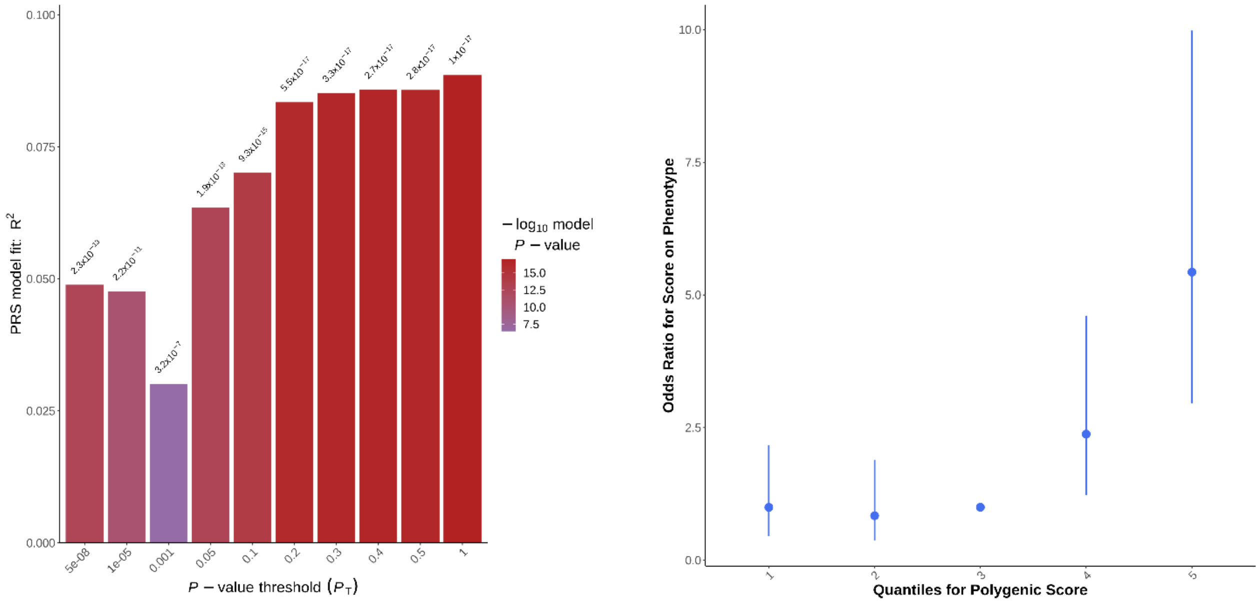

Supplementary Results Figure 11. Polygenic Risk Score Model – no chromosome 6. Bar chart x-axis shows the PRS at different levels of GWAS significance; y-axis shows proportion of the phenotype accounted for by the model. The significance of each model is shown on top of each bar. Dot plot shows the odds ratio of developing the phenotype at each quantile.

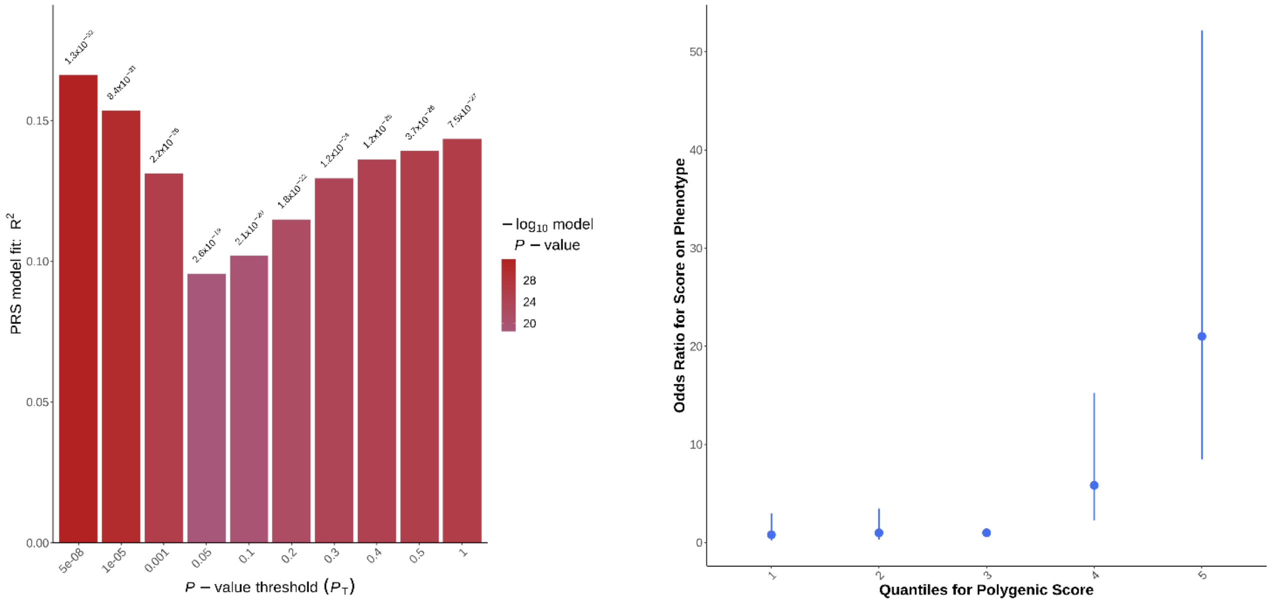

Supplementary Results Figure 12. Polygenic Risk Score Model – MAF 0.05. Bar chart shows the PRS with different levels of GWAS significance on the x-axis and the proportion of the phenotype accounted for by the model on the y axis. The significance of each model is shown the p-value on top of each bar. Dot plot shows the odds ratio of developing the phenotype at each of five quantiles of the PRS.

Supplementary Results Figure 13. STRING database plot. Starting with LGI1 and PTPRD as seed proteins, and restricting analysis to the first 20 interactors with high confidence (0.700), STRING (on: 03/01/2024) derived a network compatible with functional connections between these two entities, with a PPI enrichment p-value < 1.0e-16, indicating above-expected number of interactions for a comparable random set of proteins from the genome.

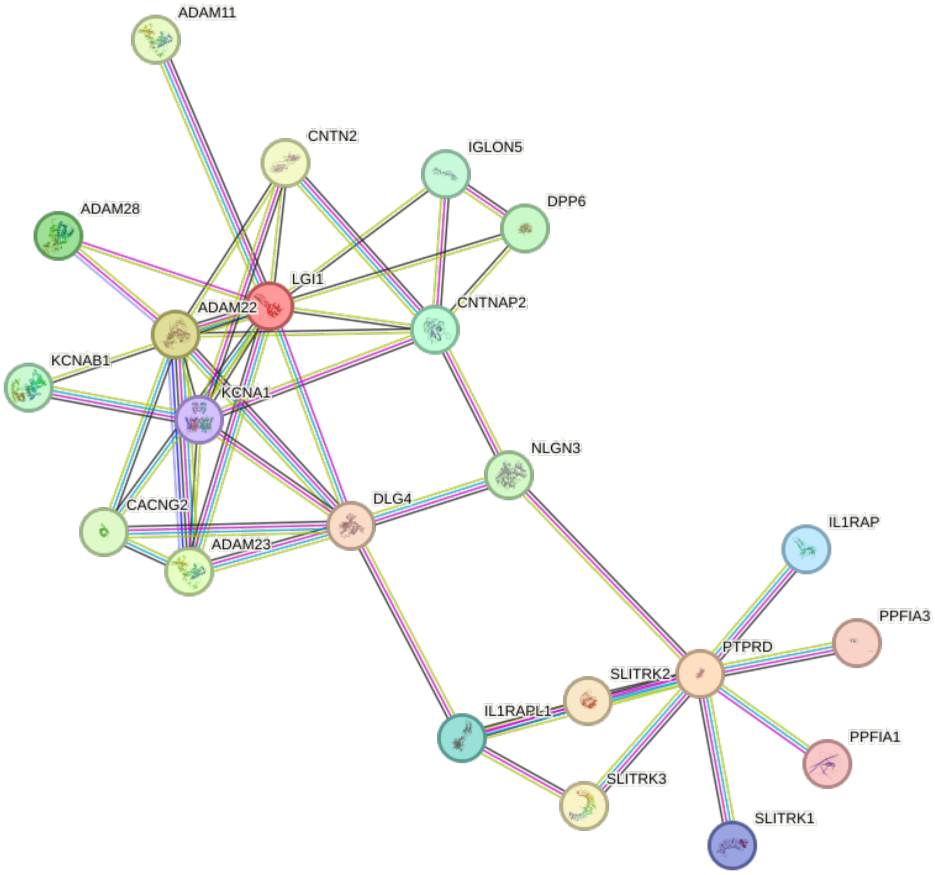

Supplement: awae349_Supplementary_Data [file awae349_supplementary_data.pdf]
